# SEARCH: Spatially Explicit Animal Response to Composition of Habitat

**DOI:** 10.1371/journal.pone.0064656

**Published:** 2013-05-22

**Authors:** Benjamin P. Pauli, Nicholas P. McCann, Patrick A. Zollner, Robert Cummings, Jonathan H. Gilbert, Eric J. Gustafson

**Affiliations:** 1 Department of Forestry and Natural Resources, Purdue University, West Lafayette, Indiana, United States of America; 2 Minnesota Zoo, Apple Valley, Minnesota, United States of America; 3 Rancho Deluxe Consulting, Stevens Point, Wisconsin, United States of America; 4 Great Lakes Indian Fish and Wildlife Commission, Odanah, Wisconsin, United States of America; 5 USDA Forest Service, Northern Research Station, Rhinelander, Wisconsin, United States of America; University of Florida, United States of America

## Abstract

Complex decisions dramatically affect animal dispersal and space use. Dispersing individuals respond to a combination of fine-scale environmental stimuli and internal attributes. Individual-based modeling offers a valuable approach for the investigation of such interactions because it combines the heterogeneity of animal behaviors with spatial detail. Most individual-based models (IBMs), however, vastly oversimplify animal behavior and such behavioral minimalism diminishes the value of these models. We present program SEARCH (Spatially Explicit Animal Response to Composition of Habitat), a spatially explicit, individual-based, population model of animal dispersal through realistic landscapes. SEARCH uses values in Geographic Information System (GIS) maps to apply rules that animals follow during dispersal, thus allowing virtual animals to respond to fine-scale features of the landscape and maintain a detailed memory of areas sensed during movement. SEARCH also incorporates temporally dynamic landscapes so that the environment to which virtual animals respond can change during the course of a simulation. Animals in SEARCH are behaviorally dynamic and able to respond to stimuli based upon their individual experiences. Therefore, SEARCH is able to model behavioral traits of dispersing animals at fine scales and with many dynamic aspects. Such added complexity allows investigation of unique ecological questions. To illustrate SEARCH's capabilities, we simulated case studies using three mammals. We examined the impact of seasonally variable food resources on the weight distribution of dispersing raccoons (*Procyon lotor*), the effect of temporally dynamic mortality pressure in combination with various levels of behavioral responsiveness in eastern chipmunks (*Tamias striatus*), and the impact of behavioral plasticity and home range selection on disperser mortality and weight change in virtual American martens (*Martes americana*). These simulations highlight the relevance of SEARCH for a variety of applications and illustrate benefits it can provide for conservation planning.

## Introduction

Individual-based (or agent-based) modeling is established as a valuable approach in disciplines such as landscape ecology and conservation biology for cases where individual variation and behavior are important drivers of system behavior [1], [2]. The conceptual basis for individual-based modeling is that the behavior of higher-level aggregations (i.e. populations, communities, ecosystems) can be simulated through the mechanistic behavior of the individuals that comprise that system [3]. In such systems the population-level attributes of simulated species emerge from the behavior and interaction of individuals that act according to detailed mechanistic rules [1], [4]. This bottom-up approach offers the opportunity for the marriage of traditional behavioral ecology (which focuses on the small-scale behavior and individual variation) and population and community ecology (which focus on large-scale dynamics of higher-order groups) through the field of behavioral landscape ecology [5]–[7].

Early IBMs used simplified behavioral rules for simulated animals in order to reduce computing time and reduce model complexity [8]. Unfortunately, oversimplified behavioral rules have persisted in many modern models that are less constrained by computing power. IBMs typically still include simplifications of animal behavior such as fixed dispersal distances, omniscient dispersers, or purely random walks [5], [9], [10]. The omission of behavioral complexity can have important implications. Research has shown that complex behavioral decisions drive patterns of animal movement [5]. Empirical studies show that movements based on behavioral decisions drive population expansion [10], species invasion success [11], and animal response to changing landscapes [12]. Further, ecological modeling has demonstrated that complex behavioral decisions can dramatically affect the viability of populations [13] and metapopulations [14], [15].

There is a need to incorporate more behavioral complexity into IBMs to understand the main drivers of animal dispersal and population dynamics [16]–[19]. Models that include too much detail, however, are in danger of becoming overly convoluted and difficult to interpret [20]. Therefore, models should strive for the optimal degree of complexity [20]. Because the appropriate degree of complexity is difficult to assess and may vary with the research goal, models with user-controlled levels of complexity are ideal. Individual-based models that are able to include sufficient behavioral complexity allow for the creation of a virtual environment in which a wide variety of questions related to population ecology could be investigated at relatively low cost and considerably lower effort than large scale field experiments.

One area of research that is well suited for individual-based modeling is animal dispersal. Natal dispersal [21]–[23] is vital for the maintenance of viable populations because it is associated with reductions in inbreeding [24], expansion of population range [25], the “rescue” of metapopulation patches [26], reduction in intraspecific competition for resources and mates [27], and the ability of wild populations to respond to dynamic landscapes [28]. Dispersal constitutes a complex interaction between landscape characteristics and animal behavior. Due to the rarity of dispersal events and the challenges associated with observing it, empirical research on animal dispersal is difficult [29], [30]. However, individual-based modeling offers a method for investigating the process and effect of animal dispersal [31].

Here we present program SEARCH (Spatially Explicit Animal Response to Composition of Habitat), a spatially explicit, individual-based model that incorporates a great degree of behavioral complexity. SEARCH simulates the dispersal of animals across a virtual landscape comprised of vector-based Geographic Information System (GIS) maps that determine the movement, foraging, mortality and spatial arrangement of animals (referred to as movement, food, risk and suitability maps, respectively). These maps can represent real areas such as those generated from remotely sensed data or theoretical landscapes with particular characteristics like those created based upon habitat metrics. These maps are vector-based rather than raster or regular geometry to reduce the potential for bias from spatial representation [32]. As animals move they respond to local stimuli such as habitat boundaries and are capable of changing behavior states (e.g. foraging vs. searching) in response to their experience. Simulations in SEARCH may employ temporally dynamic landscapes, such that the environment to which animals respond changes during the simulation. Thus, simulations can model events such as land cover change, succession, seasonal shifts in food availability, or diurnal patterns in predation pressure. As SEARCH is a population model and incorporates breeding stochastically, simulations may span many years and population-level trends emerge as a result of the behavior of the individuals. SEARCH also has flexibility in that many components can be easily turned on or off allowing for variation in model complexity. All code for SEARCH was written in the C# language utilizing the.NET framework and employs ArcGIS (Environmental Systems Research Institute, Redlands, California, USA) for map manipulation procedures. SEARCH is freely available in many versions (as a graphic user interface or command-line application) at http://code.google.com/p/paz-search.

SEARCH incorporates concepts and features from numerous other individual-based and spatially explicit models. The modeling work of Gustafson and Gardner [33] and Gardner and Gustafson [25], which simulates animal movement along with energetics and predation of dispersers, provide an important conceptual foundation for this model. SEARCH also incorporates aspects and concepts of other simulation models including (but not limited to) the dynamic landscapes of ALMaSS [34] and BACH-MAP [35], vector-based movement of Vuilleumier and Metzger [30], behavioral state changes of Morales et al. [36], boundary permeability of HexSim [37] and habitat selection rules similar to Kramer-Schadt et al. [38] and Wiegand et al. [39]. SEARCH incorporates many features of the models listed above (and others) into a single population model which allows researchers to include a high degree of behavioral complexity and landscape dynamics. Most features in the model are optional and most users would not use all capabilities of SEARCH in a single simulation, rather, model complexity would be driven by the research question. In fact, SEARCH incorporates flexibility to the degree that it could be considered a modeling framework in which alternative features can be implemented to create particular models.

To illustrate the ways in which the added behavioral complexity of the program may be utilized, we present examples of the application of SEARCH to three study populations. We demonstrate map-swapping capabilities of SEARCH and illustrate its use to investigate the impact of seasonally dynamic food resources on raccoon (*Procyon lotor*) dispersers. Similarly, we demonstrate the impacts of temporally variable predation risk on eastern chipmunk (*Tamias striatus*) dispersal. Additionally, the effects of behavioral state changes on weight distribution and disperser mortality were investigated using American martens (*Martes americana*). Finally, we simulated virtual martens that were given a range of home-range choice rules that varied the importance of food resources, safety, proximity, and search time when selecting a home-range center to determine their effect on settlement time, dispersal distance, and disperser mortality. Through these simulations we were able to highlight some of the capabilities of SEARCH and underscore the importance of incorporating an appropriate but flexible degree of behavioral complexity into individual-based models.

## Model Overview

The following model description follows the Overview – Design Concepts – Details (ODD) protocol for describing individual-based models [40], [41]. Therefore, the “Model Overview” section describes the model in broad terms, the “Design Concepts” section discusses certain design concepts and the degree to which they are employed in SEARCH, the “Details” section and the supplementary materials give detailed descriptions of the processes and algorithms used in the model and the remaining sections describe case studies conducted to illustrate the capabilities of SEARCH.

### Purpose

SEARCH simulates the dispersal and home-range establishment of animals across a virtual landscape. Animals respond to four vector GIS layers that contain values used by the rules for animal movement, foraging, risk of predation, and the suitability of habitat for home-range establishment and the configuration of areas occupied by established resident animals. Thus, users are able to investigate such factors as the potential impacts of landscape change, habitat permeability, and energetic budgets on animal populations. Output from SEARCH provides information on both the characteristics of animal dispersal as well as the associated emergent population-level attributes. SEARCH can be used to simulate a variety of species and utilizes research from disparate fields such as animal movement, foraging ecology, and physiology to parameterize the model.

### State Variables and Scales

Individuals within SEARCH can be one of two classes – juvenile dispersers or adult residents. Dispersers are characterized by a unique number, sex, weight, perception, activity mode, behavioral state, and location. State variables for residents include animal number, sex, and home-range location. Population characteristics such as age structure, sex ratio, and mortality rates can be derived from these model outputs.

Dispersers interact with the environment by responding to habitat characteristics represented in four vector GIS maps. Polygon values contained in these maps drive animal movement, foraging, mortality and home-range establishment by dictating correlated random walk movement parameters, the probability of acquiring prey and size of prey, the probability of being killed, and the suitability for home-range establishment and whether or not a location is currently occupied by a resident animal, respectively (Table 1).

**Table 1:** Landscape parameter maps and field definitions input by the user to reflect variation in animal behavioral or physiological responses to different GIS classifications.

Map type	Field	Definition	Range
Movement	Tortuosity	Tortuosity of movement drawn from a wrapped Cauchy distribution where 0 produces a purely random walk and 1 produces a linear movement	0–1
	Step length	Mean step length (m) per time-step; includes a field for standard deviation (±SD)	≥ 0
	Energy use	Energy used per time-step	≥ 0
	Crossing	Rank of location quality	≥ 0
	Perceptual window modifier	Modifies distance of perceptual window	≥ 0
Food	Probability	Probability of capturing a prey item per time-step	0–1
	Size	Mean size energy gain of captured prey; includes a field for standard deviation (±SD)	≥0
Risk	Probability	Probability of mortality due to depredation per time-step	0–1
Suitability	Suitability	Suitability of habitat for home-range establishment	0 or 1
	Occupancy	If occupied by a male	0 or 1
	Occupancy	If occupied by a female	0 or 1
Release		Location, number, and gender of animals upon initialization	na

Time periods in SEARCH are discrete. The user defines the time-step length (≥ 1 minutes), dispersal season length (≥ 1 day), and the number of years of simulation (≥ 1 year). The period of the year outside of the dispersal season (i.e. the inter-dispersal period) is modeled as a single, discrete time period. Spatial scales employed by SEARCH follow the resolution and extent of user-input GIS maps. The extents for case studies presented below are 0.25 km^2^–660 km^2^ but larger areas can be used [42].

### Process Overview and Scheduling

In SEARCH, the user parameterizes the model to emulate the movement, foraging ecology, and habitat use of the study species (Table 2). Additionally, the default behavior of individuals can be modified based upon sex, behavioral state, and time (Table 3).

**Table 2:** Animal parameter values input by the user for the temporal aspects of the simulation along with basic attributes of virtual animals.

Parameter	Description
Dispersal season dates	Start and end dates for dispersal season each year and the number of years to conduct simulation runs
Time-step resolution	Number of minutes between time-steps
Start time	Time of day that dispersal begins
Activity and resting periods	Hours (±SD) of activity and rest from start time
Home-range center selection threshold	Number of steps or suitable and unoccupied sites traversed before selecting a home-range center
Minimum home-range size	Gender-specific minimum area required for a home range
Distance weighting factor	Gender specific coefficient that modifies the effect of proximity on home-range center selection
Energy	Initial, minimum and maximum energy allowable for each animal. Death occurs below the minimum value
Search/forage trigger	Energy threshold below which animals switch behaviors from primarily searching to primarily foraging
Perception window	Distance at which habitat suitability and occupancy are perceived during dispersal
Safe to risky	Probability of switching from safe behavior to risky behavior
Risky to safe	Probability of switching from risky behavior to safe behavior

**Table 3:** Modifiers of animal behavior employed in SEARCH which allow the user to modify habitat values by multiplying them by a real number to reflect variation caused by gender, activity mode, vigilance mode, time of day, and date.

Modifier	Parameter modified
Prey acquisition probability	Probability of acquiring prey in a given GIS classification
Predation probability	Probability of mortality due to predation in a given GIS classification
Energy use	The energy used in each GIS classification
Movement speed	The movement distance per time-step in each GIS classification
Movement tortuosity	The movement tortuosity in each GIS classification
Perception	Animal perception distance in each GIS classification

During SEARCH simulations, animals traverse a virtual landscape comprised of four GIS maps that each contains multiple parameter values that are used to model virtual animal behavior (Table 1). These maps are user created and can represent biologically relevant landscape features such as habitat type, land use, land cover, or topology. The initial landscape is populated by adult residents and/or released juveniles from the social and release maps, respectively. The initial population is input by the user depending on the scenario that best models the system under study. As animals move throughout the landscape, all four maps are queried during each time-step by each animal. After each animal movement segment, the location, energetic reserves, and behavioral states are updated for that individual. Animals move, forage, die and establish home ranges according to habitat and species parameters. During a time-step, each animal completes every action for that time-step in sequential order based on animal number assigned geographically at the beginning of each year.

Once a user-defined threshold for number of steps taken or number of sites visited is exceeded, animals select a site for a possible home-range center from areas searched during dispersal. Virtual animals then move to that site, and attempt to establish a home range but may continue dispersing if they fail to locate a site with unoccupied suitable habitat. Animals become residents once a home range is established, but die if a home range is not established during the dispersal period. During the inter-dispersal period, residents are subject to random mortality and females stochastically give birth to young which disperse the following dispersal season.

## Design Concepts

### Objectives

In SEARCH each disperser's objective is to establish a home range before the end of the dispersal period. The behavioral traits of virtual animals are expected to affect dispersal success. Animals can be parameterized to remain in habitat that is of higher quality than adjacent areas. Since habitat quality in simulations is typically parameterized to correlate with increased safety and foraging opportunities (although it need not be) dispersers that remain in higher quality areas are typically less susceptible to predation or starvation. Similarly, animals that switch behavior due to perceived danger or low energy reserves behave in a way that minimizes risk of predation or starvation. Finally, implicit in many of the home-range selection criteria is the assumption that animals choosing home-range locations of higher quality (i.e. better foraging opportunities and/or lower mortality risks) will have offspring that are less likely to succumb to predation or starvation and will therefore have increased fitness.

### Adaptation

Simulated animals make decisions in response to the environment and change behavior based on their individual experiences. Such dispersal behaviors are expected to change dispersal success. For example, animals calculate whether to cross a habitat boundary during dispersal by comparing the relative habitat value at their current location with adjacent locations based upon values in the movement map. Implied in this decision making is a simple predictive model that assumes that remaining in areas of higher habitat quality will increase their chance of home-range establishment. Animals also evaluate the probability of successfully capturing prey/forage when selecting a location for a home range. In addition, animal activity mode may change based on a user-defined energy threshold and the behavior of active animals can also be affected by their perceived risk of predation. In these ways, virtual animals respond to perceived danger or low energy reserves by changing their behavioral state to respond to conditions.

### Sensing

Animals in SEARCH are able to detect information in their environment and respond to that information accordingly. During movement, virtual animals sense the predation pressure, foraging resources, habitat quality and the suitability of habitat for home range establishment along with the presence of resident animals around them. These attributes are detected by virtual animals at a distance dependent upon the perceptual window of that animal (see S.1.4 of Text S1 for detail). The characteristics detected by virtual animals can be influenced by their behavioral states, energetic reserves, gender, time of day and season such that animals maintain a memory of the habitat as they perceived it during dispersal. If animals revisit a location, the most recent memory is stored and the totality of this memory is used when animals select potential home range centers.

### Interaction

SEARCH incorporates little interaction between individuals and no direct interaction between dispersers. Indirect interaction between individuals is modeled through the restriction of non-overlapping home ranges between same-sex animals. This implies some form of interactive exclusion between individuals. Similarly, inter-dispersal reproduction implies male-female interaction though this is not modeled explicitly in SEARCH.

### Stochasticity

Nearly every action taken by animals in SEARCH is probabilistic, thus stochasticity is critical. Animals in SEARCH move based upon a correlated random walk where path tortuosity can vary from completely random (mean vector length = 0) to completely straight (mean vector length = 1) based upon a value drawn from a wrapped Cauchy distribution [43], [44] that the virtual animal obtains from the polygons in the movement map. In addition, when dispersing individuals calculate the probability of crossing a habitat boundary they do so by comparing the quality values of adjacent polygons in the movement map to a random number to determine whether to cross a habitat boundary.

Animal foraging uses stochasticity by assigning a probability of successfully foraging to each polygon to determine if an animal gains energy. The amount of energy gained during successful foraging bouts is drawn from a normal distribution based on a user-specified mean and standard deviation derived from a map polygon from the food map.

The likelihood of an animal dying during a time-step is assigned based on the animal's location, the time of day, season, and animal parameters. Stochasticity is incorporated through a randomly drawn number that determines if that animal dies. Resident mortality is calculated in a similar way, although resident morality probabilities are aspatial and occur during each time-step of the dispersal season as well as once during the inter-dispersal season.

The vigilance mode of dispersing animals is determined by a stochastic perception of risk. In this way, animals switch between risky and safe behaviors. Animal activity bouts (i.e. active/resting) are also determined by a stochastic process. The duration of each active and resting period is drawn from a normal distribution based on a user-defined mean and standard deviation of time.

Home-range center selection is weighted toward higher quality sites but incorporates stochasticity in selecting a location. Animals create home range polygons by generating a set of points around a potential home-range center with random orientation and with distances that are drawn from a distribution based on the minimum home-range area for each sex to delineate the vertices of that home-range polygon.

Breeding incorporates a number of stochastic processes. Whether a resident female becomes pregnant during an inter-dispersal period is based upon the probability of a female breeding. For those female residents that do become pregnant, the number of offspring produced is drawn from a normal distribution based on a user-specified mean and standard deviation of litter size. The sex of each offspring is determined probabilistically according to the sex ratio parameter.

### Emergence

Population-level patterns emerge based upon the behavior and interactions of the individual animals in response to the spatio-temporal configuration of habitat and conspecific residents. These emergent properties develop from the interaction of baseline behavioral parameters, individual variation (due to gender, activity mode, etc.), interaction between individuals and stochasticity. Such higher-order emergent properties can include population density and spatial configuration, animal weight distribution, mortality rates by source (starvation vs. predation), and mortality locations.

### Observation

During SEARCH simulations, data on the behavioral state (energetic, vigilance mode, etc.) and fate of each individual are produced during every time-step. This output includes a GIS polygon map that depicts the animal movement and perception during dispersal. Following each dispersal season, a landscape map is also produced that depicts all existing home ranges. Other population-level attributes (such as annual survivorship, population density, habitat selection, etc.) can be calculated from the individual and population output.

## Details

### Initialization

SEARCH can be initialized to reflect one of three scenarios. First, the simulation can begin without existing home ranges and all dispersers can be created based upon a map of releases. Such a simulation could model the reintroduction of a species into an area from where it had been extirpated. Second, the simulation can begin with established home ranges throughout the landscape without any released animals. Thus, all dispersers would be the result of reproduction of established females (chosen randomly based upon user-input parameters). This scenario would best reflect the population dynamics of an established population. Finally, simulations can implement a combination of resident reproduction and release of individuals. In this way an augmentation or supplementation of an existing population can be modeled [42].

At the beginning of each simulation an empty memory map is created for each individual. This map reflects the area perceived by an animal during dispersal and the occupancy and suitability of all areas observed. During each time-step the memory map is updated for every animal to reflect the area perceived during dispersal. When animals begin selecting potential home-range centers, this map is used to eliminate all points in areas perceived as unsuitable or occupied. For each animal a text file is also created that records the animal's conditions for all state variables (i.e. location, energy level, etc.) during each time-step.

### Input

#### Time Parameters

SEARCH incorporates flexibility in the temporal scale and extent of simulations. The user inputs the start date of the simulation, the number of years to simulate (≥ 1), the number of days in the dispersal season (≥ 1), the start time of day 1 of the dispersal season (0–23 hours) and the length of each time-step in minutes (≥ 1).

#### Maps

At the most basic level, virtual animals in SEARCH respond to the user-specified parameters of five GIS maps (Table 1). These map types include 4 polygon maps (movement, food, risk and suitability) and 1 point map (assigning location and number of released animals). The landscape in SEARCH is dynamic in that any of the 5 GIS layers can be replaced at any time during the simulation with a map with different parameters and/or spatial configuration. Such map swapping can be employed to simulate habitat change or management, landscape disturbance or simply the variation in animal response to habitats at different times of day or seasons (see case studies for examples).

#### Species Attributes

Energy parameters (with no units) represent the initial energetic reserves of each disperser, the minimum allowable energy level (below which animals die of starvation) and the maximum possible energy level (Table 2). During each time-step, energy is lost based upon the energetic cost associated with a particular habitat as defined in the movement map. Energy is gained if prey or forage is acquired based upon the probability of foraging success and the amount gained is a function of the size of the item on the food map.

Active dispersers move across the landscape relative to the various parameters on the movement map (resting animals remain static). The mean durations of active and resting periods are assigned by the user (along with a standard deviation) that applies to all dispersers. Activity periods of individual animals, however, may diverge based upon the stochasticity in period length due to variance around the mean (described by the standard deviation) so that animal activity and rest cycles need not be synchronized with one another. Animals may have many active and resting periods within a single day but must begin each year active. The mean values of all active and rest periods must sum to 24. Variability around mean active and resting periods may cause animals to have activity periods that do not exactly follow a 24-hour cycle.

Within SEARCH, active, dispersing animals can be either foraging or searching. The particular behavior of each activity mode can be specified by the user through the parameterization of modifiers (see “Modifiers” in “Details” section; Table 3). The user could parameterize SEARCH so that the probability of an animal successfully capturing a forage or prey item, for instance, would be higher during foraging activity but lower when in searching mode. All animals begin in searching mode but switch to foraging if their energetic reserves fall below a user-defined threshold. Note both searching and foraging animals are both capable of foraging or establishing home ranges.

As with activity modes, virtual animals can also exhibit one of two possible vigilance modes at any time. Individuals can either be in safe mode or risky mode. The modifiers (as defined by the user; Table 3) that affect animal behavior can differ based upon an animal's vigilance mode. The risk of an animal being killed, for instance, could be decreased when an animal is in safe mode (reflecting higher vigilance, for example) relative to risky mode. Animals begin each dispersal season in risky mode but will change to safe mode during any time-step if a randomly drawn number falls within a user specified interval (see section S.7.2 of Text S1 for details). Animals in safe mode change back to risky mode if a randomly drawn number falls within the user specified interval (see section S.7.2 of Text S1 for details).

The individual memory of each animal in SEARCH is retained explicitly through the use of a memory map. An animal's perceptual range is the distance at which it can perceive and respond to landscape features [45]. SEARCH, however, utilizes a perceptual window that includes both perceptual range and small scale wandering of animals during a time-step (similar to assessment corridors of Doerr and Doerr [46], ellipses of Bélisle et al. [47], and circle-ellipses of Selonen et al. [48]. In SEARCH, this perceptual window is the area (with radius in meters) animals perceive during dispersal. Within this memory map, animals retain a complete record of the suitability and occupancy of perceived areas. If a specific location or area is revisited, the most recent suitability and occupancy status is overwritten on the memory map. The perceptual distance of an animal can be modified based upon that habitat type in which an animal occurs, the time of day, or the season. For example, the user can reduce the animal-perception window during low moonlight relative to the full moon perception window [49].

#### Home-Range Attributes

Animals in SEARCH can be triggered to begin selecting home-range centers based upon one of two user-selected criteria – either a user-specified number of time-steps have elapsed since the inception of dispersal or the animal has visited a user-specified number of suitable and unoccupied sites. Once this trigger is surpassed, animals choose their preferred home-range center based on user-specified criteria including the factors that most influence patch selection [50], [51]. These criteria consider either (1) the proximity of the site (“closest”), (2) the proximity and food availability of a site (“food”), (3) the proximity and risk of mortality at a site (“risk”), or (4) the proximity, food and risk of a site (“integrated”). The user chooses one of these four criteria for each run. The user also inputs home-range establishment requirements including the minimum area for a home range for each sex and the relative importance of site proximity in home-range center selection for each sex (Table 2). It is possible for users to effectively negate the effect of proximity in home site selection by choosing a large value for the distance weighting factor (Table 2).

#### Modifiers

In SEARCH, the user can modify the baseline parameters for many behavioral traits of animals to reflect the variability in behavior as a result of an animal's gender, behavioral state, the time of day, or the season (Table 3). Modifiers can be created for both sexes (male and female), all four behavioral states (risky-searching, risky-foraging, safe-searching, and safe-foraging), and any number of temporal modifiers at two scales (hourly, daily). For instance, the perceptual distance of an animal that relies on vision may be increased during the day relative to night [49].

#### Resident Attributes

Residents are assigned a single, user-specified probability of mortality during each time-step (independent of location) but during the inter-dispersal period are subject to a single mortality probability. Additionally, a proportion of randomly selected females give birth to a user-defined (mean±SD) number of young (when this integer is negative, 0 is used) that have a sex ratio based on the defined probability of female offspring. Young begin dispersal in the subsequent season at the center of the mother's home range.

### Submodels

See Supplementary material for details including submodel descriptions (Text S1), technical documentation (Text S2) and process flow diagrams (Figure S1).

## Illustrative Case Studies

To demonstrate the functionality of map swapping, behavioral states and home range selection in SEARCH, we provide a number of case studies here. These case studies are meant to be illustrative of the capabilities of the model rather than complete studies of model performance. These examples highlight some of the possible applications of SEARCH to real-world scenarios and demonstrate SEARCH's applicability to a variety of systems.

### Case Studies: Food Map Swapping

SEARCH is unique among individual-based models in that it allows the user to exchange any of the four GIS maps to which virtual animals respond (i.e. movement, risk, food, or social) at a daily, seasonally, or yearly time scale allowing temporally dynamic parameters. To illustrate the functionality and implications of these map swapping capabilities we simulated two study systems (raccoons in Indiana and chipmunks in Wisconsin).

In the first study system, the effects of seasonally dynamic food resources on raccoon population dynamics were investigated. Raccoons in north-central Indiana experience dramatic shifts in forage availability throughout the year due to corn maturation and the subsequent superabundance of food resources [52], [53]. This temporal shift in food availability was modeled in SEARCH by swapping food maps during corn maturation in the summer to reflect the increase in both the likelihood of successful foraging and the amount of energy gained during a foraging bout in an agricultural polygon. Because agriculture was the dominant cover type in the area and virtual raccoons were expected to forage heavily there, we predicted that raccoons that experienced temporally dynamic food resources would have dramatically different changes in mass compared to animals exposed to static foraging resources.

#### Methods

Raccoon simulations were conducted for a single year with a dispersal season of 150 days (the maximum observed raccoon colonization time in one study – Beasley unpublished data) and 1 hour time-steps on GIS layers digitized from USGS aerial photos of a 4 km×4 km area of the upper Wabash River basin in north-central Indiana (for more detail see: [53] and [54]; Supplementary material, Table S1) simulations began with resident raccoons present on the landscape based on estimated raccoon density in the study area [55] and all dispersers were the product of resident reproduction. For virtual raccoons, energy parameters were used as a surrogate of mass. To approximate raccoon weight limits observed in field studies, dispersers began with a mass of 3750 g, had a maximum mass of 10000 g and died of starvation if their body mass fell below 1800 g [56], [57], (Beasley unpublished data). Dispersers were active for 12 h during the night (1800 until 600) and rested for 12 h during the day [58], [59], [60]. Translocated raccoons typically establish home ranges after 2 weeks of dispersing [61] so this value (168 active steps) was used as the trigger time for virtual raccoons to begin choosing home-range centers. Animals used the integrated criteria for home-range center selection and had minimum home-range sizes of 0.29 km^2^ and 0.12 km^2^ for males and females, respectively [53]. Resident virtual raccoons were subject to a mortality probability each time-step (8.34×10^−5^) and during the inter-dispersal season (0.194) based on published raccoon survival estimates [62]–[66]. Surviving resident females had a 90% pregnancy probability during the inter-dispersal season [67], [68], Beasley unpublished data) and produced young based on a litter size with mean 3.5 and standard deviation of 1 [56], [69]–[72], Beasley unpublished data). To simulate corn maturation, virtual animals were exposed to maps with low food resources in agricultural areas for 37 days, superabundant food resources in these areas for 76 days and then low resources for another 37 days. The weight distribution of successful dispersers in simulations with dynamic food maps was compared to that of the null model with a constant intermediate forage probability and energy gain. All modifiers were set to 1 to effectively eliminate variability due to gender, time, or behavior.

For each scenario, 10 replicates were conducted and the weight change of every successful disperser in each simulation was recorded. Because the weight changes of dispersers within a simulation were not independent, we used a mixed modeling approach to avoid pseudoreplication [73], [74]. Each replicate simulation was nested within the corresponding scenario (static or dynamic food resources) and a nested ANOVA was conducted [75] to determine if the mean weight change of virtual raccoons differed among the scenarios.

#### Results/Discussion

**Figure 1:**
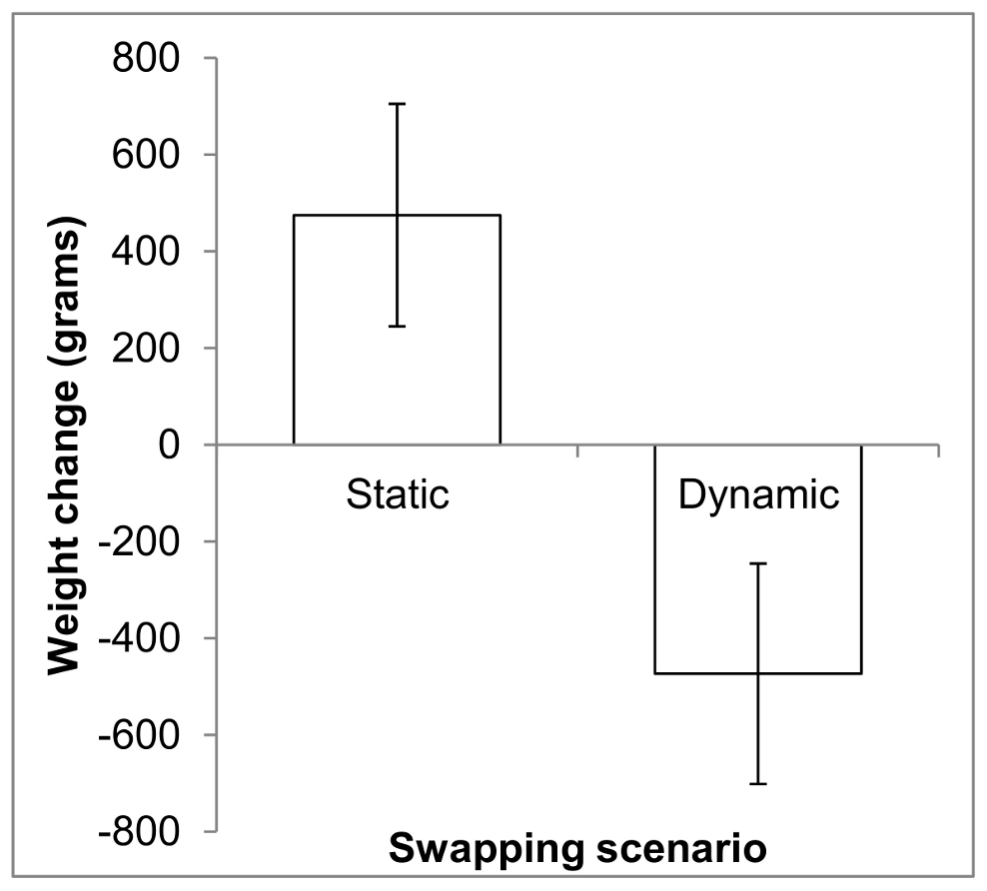
Raccoon weight change. Mean (± 1 SD) change in weight of virtual raccoons for simulations with static and temporally dynamic food maps. Weight change values are for animals that successfully established home ranges during the simulation (Base n = 58; Swap n = 54).

In simulations of raccoons, the weight distributions of animals differed significantly between simulations with and without temporally dynamic food resources (t_18_ = −20.78, p<0.0001). Virtual raccoons that experienced static values for energetic gain from agricultural habitats gained an average of 475 g (SD = 230, n = 58). Virtual raccoons subjected to temporally dynamic agricultural food resources (with relative scarcity followed by superabundance and then scarcity again) lost an average of 473 g (SD = 228, n = 54) during the simulations (Figure 1).

The observed differences in virtual raccoon weight changes seem to be due to the amount of time animals dispersed before settling. Nearly all (96%) successful dispersers in the temporally dynamic simulation settled before agricultural areas produced superabundant food resources. These animals, therefore, were only subjected to food scarcity in agricultural areas and, not surprisingly, all lost weight during the simulation. The two dispersers that settled after the food map swap either settled one day after the food switch (losing 1108 g) or settled well after the superabundant food emergence and gained 701 g during the simulation. Therefore, temporally dynamic food resources appear to have a substantial effect on virtual animal weight distribution but this effect is dependent on the animal's dispersal time.

Simulations of raccoon weight changes in response to dynamic food resources underscore the impact dynamic landscapes can have on animal populations. Animal populations that exploit seasonal food resources (including anthropogenic or naturally ephemeral sources) can be easily simulated in SEARCH. This capability allows researchers to include more temporal complexity in foraging resources than would be possible in simulations with static food availability.

### Case Studies: Risk Map Swapping and Behavioral Response

An appreciation for the importance of spatial heterogeneity in predation pressure on animal populations (i.e. the ‘landscape of fear’; [76] has gained recognition within the ecological community. Numerous animal populations are impacted by and respond to spatial variation in mortality risk [77]–[81]. The importance of temporal heterogeneity in predation pressure has been studied less frequently, however. Temporal variation in mortality risk has been shown to have significant impact on survival and behavior in a few populations [82]–[84].

We investigated the potential population-level impacts of temporal variation in predation and the impact of behavioral response to such variation by modeling the dispersal of chipmunks in northern Wisconsin (Supplementary material, Table S2). Over the entirety of each simulation run virtual chipmunks were exposed to 1) a homogeneous static risk of mortality, 2) a spatially heterogeneous but temporally static predation risk or 3) different diurnal and nocturnal predation probabilities that were both spatially heterogeneous. Additionally we simulated virtual chipmunks that responded to variation in predation risk through variable permeability of habitat boundaries. Animals either had no response to habitat boundaries, or preferentially remained in habitat with lower predation risk (independent of time), lower predation risk based upon current time or lower predation risk based upon predicted future risk.

#### Methods

Chipmunks are exposed to different predators at different times of day, such as raptors during daylight and mustelids at night [85], [86], (Zollner unpublished data). Furthermore, the timing of chipmunk activity in conjunction with predators has been shown to affect survival in field experiments [87]. In SEARCH, predation risk was modeled using a habitat-specific, empirically derived index of predation pressure where motion sensor cameras observed relative predation intensity of taxidermied chipmunks at different times in varying habitats (Zollner unpublished data). This was combined with published annual mortality values of chipmunks [88] to estimate simulation predation rates. Data used to calculate predation probabilities were combined, segregated spatially or segregated spatially and temporally to create predation maps that were aspatial, spatially heterogeneous or spatially and temporally heterogeneous, respectively. Temporally dynamic landscapes were simulated by swapping risk maps that represented daytime (6:00 – 18:00) and nighttime (18:00 – 6:00) mortality risk. Alternatively, chipmunks were simulated that had a constant predation risk with either aspatial or spatially variable risk.

Simulation scenarios contrasted the empirical conditions described above with a range of responses to predation risk by virtual chipmunks. Animals without response to habitat boundaries used crossing values that were identical for all habitat types except for those areas into which animals never entered (cross. value none in Supplementary material, Table S2). Virtual animals that responded to spatial variation in predation risk had crossing values that scaled boundary permeability to the relative predation risk of habitat types independent of time (cross. value base in Supplementary material, Table S2). Temporally responsive animals had crossing values that determined boundary crossing relative to time-dependent predation risk either currently (cross. value day and night relative to risk map swapping in Supplementary material, Table S2) or predictively (cross. value day and night 1 hour prior to risk map swapping in Supplementary material, Table S2).

All combinations of predation variation and behavioral response were simulated for a total of 12 simulation scenarios. Therefore, animals in particular simulations 1) responded at a coarser scale than risk was simulated (under-response), 2) responded at the same scale as risk was simulated (correct-response) or 3) responded at a finer scale than risk was simulated (over-response).

Simulation output consisted of both overall and predation-specific (e.g. only animals that died of predation rather than from a failure to establish a home range by the end of the dispersal season) mortality rates. Both rates were compared among populations of all scenarios to determine if variation in predation risk and animal response to predation pressure impacted survival of the population. We predicted that both types of mortality rate in each population would scale inversely with the degree of response of the virtual chipmunks (i.e. under-response > correct response > over-response).

All chipmunk simulations were run for 2 years with a 30 day dispersal season and 5 min time-steps on GIS maps 500 m×500 m (derived from digitized aerial photos). All virtual dispersers were produced through reproduction of resident animals present on the landscape at the beginning of the simulation based on estimated chipmunk density in the study area (Zollner unpublished data). Dispersing animals were active for 4 h beginning at 9 AM [89] and had a baseline perceptual window of 120 m [90]. Animals were triggered to begin choosing home-range centers after 1000 active steps or roughly 3 weeks of dispersal [91]. Virtual chipmunks used the closest site criteria and had minimum home-range requirements of 0.002 km^2^ for both sexes [89]. All resident females became pregnant and produced litters (mean = 4.5, SD = 0.5) and had an equal chance of male and female young [89]. We eliminated energy as a limiting factor due to insufficient data by creating ideal foraging conditions (i.e. 100% success) and no energy loss during movements. Similarly, resident mortality was eliminated and all multiplicative modifiers were set as 1 to turn off variability due to gender, time and behavior.

For each scenario, 10 replicate simulations were conducted and the overall and predation-specific mortality rates of the dispersers were determined. The data were then power transformed (raised to 0.75) in order to satisfy assumptions of equal variance and normalized residuals (Shapiro-Wilk, W = 0.985073, p = 0.2086) and an ANOVA was conducted to compare overall mortality and predation rates based upon simulated risk and animal behavior. Contrasts were then used to compare the average predation rates of simulations with varying degrees of animal responsiveness (i.e. under, correct or over-response; all tests conducted in SAS 9.3; [75]).

#### Results/Discussion

**Figure 2:**
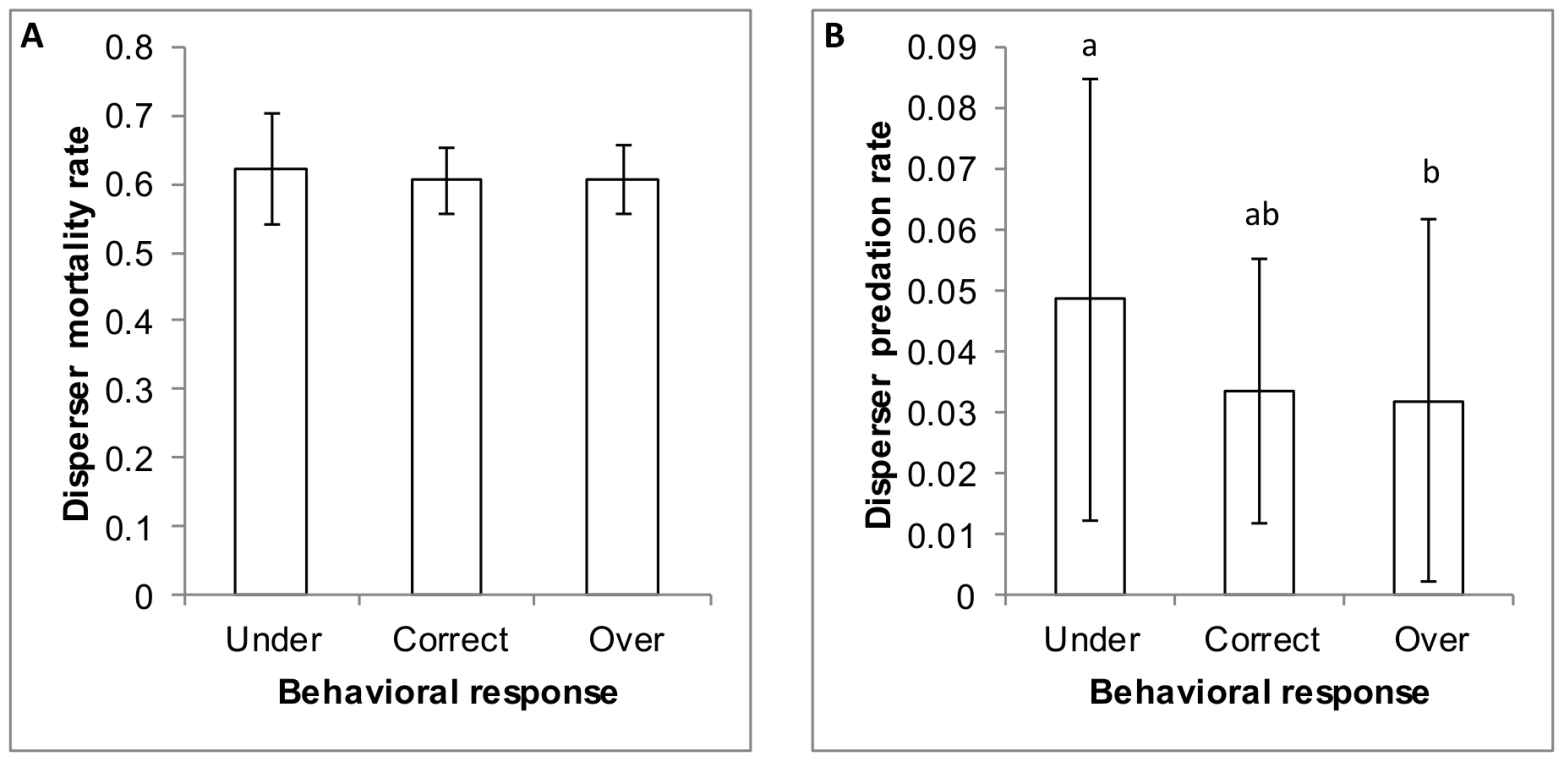
Eastern chipmunk mortality rate. Mean (± 1 SD) A) overall mortality rate and B) predation-specific mortality rate for virtual chipmunks with varying levels of response to simulated predation risk.

Simulations of virtual chipmunks suggested that the effect of predation was compensatory in the overall mortality rate of the population. Overall mortality rates did not differ among virtual dispersing chipmunks in the 12 simulated scenarios (F_11, 108_ = 0.85, p = 0.59) nor did overall mortality differ between the three levels of responsiveness of animals to their experienced predation (all pairwise p>0.25; Figure 2a). Predation-specific mortality, however, was significantly different between scenarios (F_11, 108_ = 2.08, p = 0.0277). In addition, contrasts of predation rate between levels of responsiveness differed with greater simulated mortality rates for under-responsive populations as compared to the others (Table 4, Figure 2b).

**Table 4:** Contrasts of average predation-specific mortality between simulations of virtual chipmunks with varying degrees of responsiveness to simulated predation pressure.

Contrast	Difference	Standard error	Degrees of freedom	t-value	p-value
**Over vs. Under**	−0.4425	0.1829	108	−2.42	0.0172
**Over vs. Correct**	−0.1280	0.2240	108	−0.57	0.5687
**Correct vs. Under**	−0.2772	0.1530	108	−1.81	0.0728
**Over vs. Pooled Others**	−0.5706	0.3421	108	−1.67	0.0982
**Correct vs. Pooled Others**	−0.0373	0.0818	108	−0.46	0.6493
**Under vs. Pooled Others**	0.2399	0.1002	108	2.40	0.0183

The overall mortality rates were the same for all simulations despite differences in predation-specific mortality. Thus, it appears that predation, as a result of variation in probabilities of mortality and animal response, is only a compensatory factor within overall mortality of simulated eastern chipmunks. Therefore, factors other than predation, such as competition for space, seem to be driving mortality. Interestingly, the compensatory effect of predation has also been suggested for eastern chipmunks based on the results of field studies [92].

As expected, chipmunks that were under-responsive to the variability in predation risk exhibited the highest predation rates. Interestingly, virtual animals that over-responded to predation variability had the same predation rates as those that responded at the appropriate scale. This suggests that while over-responding animals did not gain any advantage through their behavior they also suffered no mortality cost from their over-responsiveness.

These simulations highlight the capabilities and possible implications of fine-scale, temporally dynamic predation risk in conjunction with variable behavioral response in SEARCH. Most models use coarse, static mortality risk to model predation [82], [83]. SEARCH simulations with virtual chipmunks have demonstrated that the inclusion of temporally and spatially variable risk, when combined with various degrees of behavioral response, can dramatically affect predation mortality. While the overall mortality was unaffected (thus the population dynamics nearly identical) by the inclusion of this added complexity, predation-specific mortality differed greatly. Therefore, research concerned with cause-specific mortality would benefit from the fine-scale temporal component of predation available in SEARCH.

### Case Studies: Behavioral States

Many spatially explicit individual-based models are behaviorally minimalistic and assume static behavior for mathematical convenience or due to lack of empirical data [5]. Empirical research, however, has shown the behavioral states of dispersers to have dramatic effects on population dynamics [36], [93], [94]. SEARCH allows users to provide virtual animals with greater behavioral complexity by defining the conditions under which animals switch activity or behavioral state.

We conducted SEARCH simulations to investigate the impact of increasing behavioral complexity on dispersal characteristics of simulated American martens. We simulated martens with varying degrees of responsiveness to behavioral triggers (low energy reserves and narrow predator escapes) and measured the weight change of individual virtual martens along with the disperser mortality rates under each scenario.

#### Methods

Simulations were conducted for one year on GIS maps of Wisconsin (derived from data from the U.S. Forest Service – Chequamegon-Nicolet National Forest Combined Data Systems STAND data and the Wisconsin Department of Natural Resources' WISCLAND Level 3 GIS data [95]. Initial residents (17 male and 25 females) were created using the output of a separate simulation (the closest home-range selection of final case study) to approximate the distribution of martens following the four years of releases from 1987–1990 [96]. Additionally, the population was augmented with a release of 14 animals to simulate a portion of the marten releases in the study area in 2008 [97], [98].

Spatial parameters for movement were derived from marten snow backtracking data, foraging parameters were based on small mammal trapping data, and predation risk parameters were based on predator indices (Zollner unpublished data; Supplementary material, Tables S3, S4 and S5). Habitat suitability on the social map was based on Dumyahn et al. [99] and Wright [100]. The dispersal season was 60 d [101] with 1 h time-steps. Animals began active behavior at 4 am with alternating activity and rest periods of 4.5 h and 7.5 h (all with SD = 8) [102]. Marten energy values were based on conversions of body mass to kilocalories. Animals dispersed with 4548 units of initial energy and had minimum and maximum energy limits of 3866 and 5003 units, respectively [102]. Virtual martens had a baseline perceptual window of 100 m (from perceptual range of Gardner and Gustafson [25]. A baseline value of 270 active steps, or an average of 30 days, was used for a trigger value after which individuals began establishing home ranges. Virtual martens had minimum home ranges of 4.25 km^2^ and 2.32 km^2^ for males and females, respectively [99]. Residents were subject to a 5×10^−5^ time-step mortality probability and a 0.17 inter-dispersal mortality [103]. Surviving resident females had a 74.4% likelihood of becoming pregnant [104], [105], had litter sizes with a mean of 3 and standard deviation of 1 [105] with a balanced sex ratio [104]. Gender and temporal modifiers were set to 1 with the exception of the male risk modifier which was 0.7632 to model the low mortality risk of male martens compared to females [103].

We simulated virtual martens with different sensitivities to low energy reserves. Martens that fell below the energy threshold switched activity mode from searching to foraging behavior (and vice versa). Martens in foraging mode had an increased likelihood of capturing prey and a decrease in energy use, movement speed, mean vector length and perceptual window distance as compared to searching martens (Supplementary material, Table S6). The baseline, reduced and increased threshold levels for simulations were set at 4250, 4000 and 4500, respectively. We predicted a positive relationship between energetic threshold level and animal weight change due to those animals' ability to respond to their level of energetic reserves. We predicted no effect of threshold level on disperser mortality compared to simulations with behaviorally static animals, however, due to the low likelihood of animal starvation.

Similarly, we simulated martens with one of three levels of response to perceived mortality risk (animals switching from risky to safe behavior and vice versa). The baseline response was a 1% probability of switching vigilance modes during a single time-step. Animals with increased responsiveness had a 10% likelihood of changing and animals with reduced response had a 0.1% chance of changing behavior. Animals displaying safe behavior represented animals with increased vigilance and thus had an increased perceptual window [106] but decreased speed (due to vigilance pauses; [107], [108]) and mortality risk [109] compared to those exhibiting risky behavior (Supplementary material, Table S6). Because the safe-risky trigger responds only to perceived predation risk, we predicted that animals with varying levels of responsiveness would have different levels of disperser mortality but no differences in mean animal weight change over the course of the simulation as compared to virtual animal without behavioral state changes.

Finally, a scenario was conducted where marten had both a baseline level of danger response (1% probability of behavioral switch) and a baseline level of response to low energy (4250 units). Modifiers for animals in each of the four possible behavioral states were the product of the values for the activity and vigilance modes (Supplementary material, Table S6). We predicted that virtual animals with both predation and energetic behavioral responses would have different weight changes and mortality rates compared to the null model where animal behavior was constant.

Three replicates of each scenario were conducted in SEARCH. A single disperser mortality rate per simulation was recorded. Because these data satisfied the assumptions of normality (Shapiro-Wilk, W = 0.983142, p = 0.9459) and homogeneous variances (Levene, F = 2.35, p = 0.0745) the survival values for the 8 behavioral states were compared using an ANOVA. A post hoc Dunnett's comparison [110] of the control with the 7 experimental setups (three levels of risk response, three levels of energetic response, one level of both responses; simultaneous α = 0.05) was conducted.

Because the dispersal characteristics of individual animals in the same simulation were not independent (i.e. fate of one animal affected that of another such as an animal settling precluding another individual from establishing a home range in an area), animal weights were considered multiple measurements from a single replicate to avoid pseudoreplication [73], [74]. Because these data failed to satisfy both the normality and equality of variances assumptions, these data were rank transformed in order to conduct non-parametric tests [111]. The means of the rank transformed data were compared using a nested ANOVA. A post hoc Dunnett's comparison was conducted to compare the mean values of simulation runs to those virtual martens that had no behavioral modifiers relative to weight distribution (all tests conducted in SAS 9.3; [75]).

#### Results/Discussion

**Figure 3:**
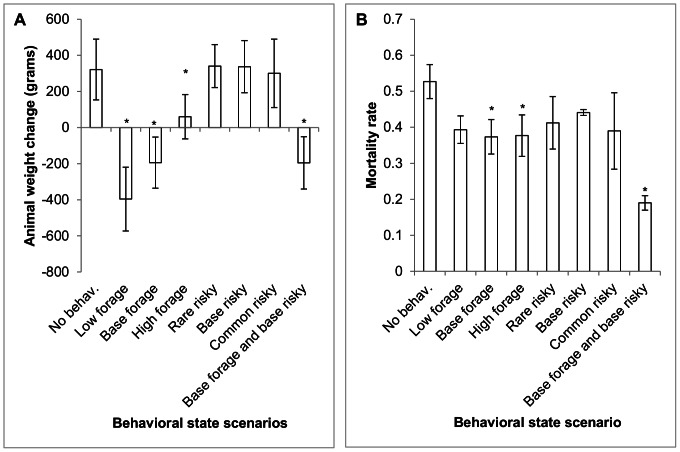
Effect of behavioral state switching on American martens. Mean (± 1 SD) marten A) weight change (pooled for all replicates) and B) mortality rate for eight behavioral state scenarios (three replicates per scenario). Simulations consisted of animals with no behavioral state changes (no behav.), a search-forage threshold of 4000 (low forage), a search-forage threshold of 4250 (base forage), a search-forage threshold of 4500 (high forage), a risky-safe proability of 0.001 (rare risky), a risky-safe probability of 0.01 (base risky), a risky-safe probability of 0.1 (common risky) or a search-forage threshold of 4250 and a safe-risky probability of 0.01 (base forage and base risky). Asterisk denotes scenarios that were significantly different from no behavior simulations.

The inclusion and degree of sensitivity of animal behavioral state changes had significant effects on dispersal mortality and weight distribution. The mean ranked weight distributions of animals differed (F_7, 16_ = 280.57, p<0.0001) and was the response variable most affected by the various behavioral states. The differences in weight changes of animals appeared to be driven primarily by the search-foraging threshold (Figure 3a). All four conditions that used the search-forage trigger (low threshold, base threshold, high threshold, base foraging threshold combined with base risky) had significantly lower weight changes compared to animals that had no behavioral state changes (Dunnett's test, p<0.05, df_error_ = 874, MS_E_ = 22968, t_crit_ = 2.6). Mean weight change had a positive relationship with forage-search threshold level where the lowest threshold level resulted in animals with the greatest weight loss. The risky-safe vigilance modes, however, had no significant effect on animal weight compared to animals without behavioral state changes (all p>0.05). There was an apparent positive correlation between the variance in animal weight and the probability of vigilance mode change though this was not tested explicitly.

As expected, the search-forage threshold had a much stronger effect on virtual marten weights than the risky-safe probability. Animals subjected to differences in foraging and searching behavior had weights approaching the threshold for behavioral state change. In these situations, animals in the searching mode lost weight until they fell below the threshold, switched to foraging mode and began gaining weight. This resulted in animal weights that commonly oscillated around the threshold value. The risky-search probability, on the other hand, was stochastic and independent of an animal's energy reserves. All levels of this parameter, therefore, had little direct effect on weight change. Instead the probability of switching vigilance modes seemed to have more influence on the variance of animal weight changes than on the mean population weight change.

Simulations including behavioral state changes differed significantly in terms of disperser mortality (F_7, 16_ = 8.10, p = 0.0003). Simulations with the high and baseline forage-search threshold as well as simulations with both the base threshold and base risky had significantly lower disperser mortality than simulations with virtual animals with static behavior (using a simultaneous α = 0.05). Most dramatically, simulations with base levels of both vigilance mode and activity mode changes had less than 40% the mortality rate of simulations with static behavior (Figure 3b).

Both types of behavioral complexity reduced mortality of virtual martens with the lowest mortality rates associated with animals with both types of behavioral response. Inclusion of the search-foraging threshold allowed animals to avoid starvation by responding to low energy reserves and changing behavior to maximize energetic gain. Similarly, the risky-safe probability allowed virtual animals to react to predation escapes and respond with safer behavior but was independent of observed mortality risk and was, therefore, purely stochastic. Thus, the forage-search threshold more dramatically affected mortality as it responded to the systematic threat of starvation while the safe-risky behavior responded to a stochastic probability of a predation escape.

Behavioral variability in SEARCH resulted in animals that behaved differently than would have been possible in simulations without this flexibility. This added behavioral complexity significantly affected dispersal characteristics of virtual martens. Therefore, the dynamics of animals that utilize different behavioral states or strategies could be dramatically impacted by the inclusion or exclusion of behaviorally complexity in simulation modeling. SEARCH allows researchers to evaluate whether this increased complexity affects the population under study and incorporates it when it is found to be necessary to accurately model the system.

### Case Studies: Home-Range Trigger and Decision Criteria

SEARCH employs a number of options for home-range selection. These selection criteria differentially prioritize sites based on the factors most associated with animal habitat selection (i.e. foraging opportunities, [112]; predation risk, [113]; proximity, [114]). Empirical and modeling studies have shown that dispersal and space use of animals is affected by how they weigh the potential costs and benefits of sites when selecting from a number of potential home range locations [39], [115], [116].

We studied the impact of varied prioritization of these costs and benefits for particular locations on dispersal distance, settlement time, and disperser mortality of virtual American martens. Dispersers in SEARCH select home-range locations based on a user-specified prioritization of attributes. We investigated the effect of the different home-range selection criteria on the dispersal of American marten. Simulations consisted of virtual animals that chose home-range locations based on only proximity (to current location), proximity and food availability, proximity and mortality risk, or proximity and food and risk together. For each scenario the dispersal distance, time to settlement and mortality of dispersers was measured. We predicted that the different home range selection criteria would result in differences in dispersal distance but not settlement time or disperser mortality because we expected animals to travel further to find appropriate home sites when using more restrictive selection criteria.

#### Methods

Marten simulations (with same parameterization as 6.3 except where specified) were run for 4 years on 33.4 km×28.7 km GIS map layers (same sources as “Behavioral States” case study). The simulation began without any resident individuals present in the area and animals were added to the simulation through releases every year that corresponded with actual releases of American martens in Wisconsin [96] as well as reproduction of successful dispersers. For each scenario, three replicates were simulated.

To satisfy the assumptions of normality and equal variances, some of the data were transformed. Dispersal distances were square-root transformed (Shapiro-Wilk, W = 0.9979, p = 0.2604; Levene, F = 1.19, p = 0.2920), settlement times were rank transformed and unmodified mortality values were used (Shapiro-Wilk, W = 0.8750, p = 0.0757; Levene, F = 1.07, p = 0.4162). Dispersal distances and times contained pseudoreplication due to the fact that the fate of an individual could be affected by that of another animal in the same simulation. Therefore, a nested ANOVA was conducted to detect differences among scenarios. Because a single mortality rate was measured for each simulation, a standard ANOVA was conducted to determine if scenarios differed in respect to disperser mortality (all tests conducted in SAS 9.3; [75]).

#### Results/Discussion

**Figure 4:**
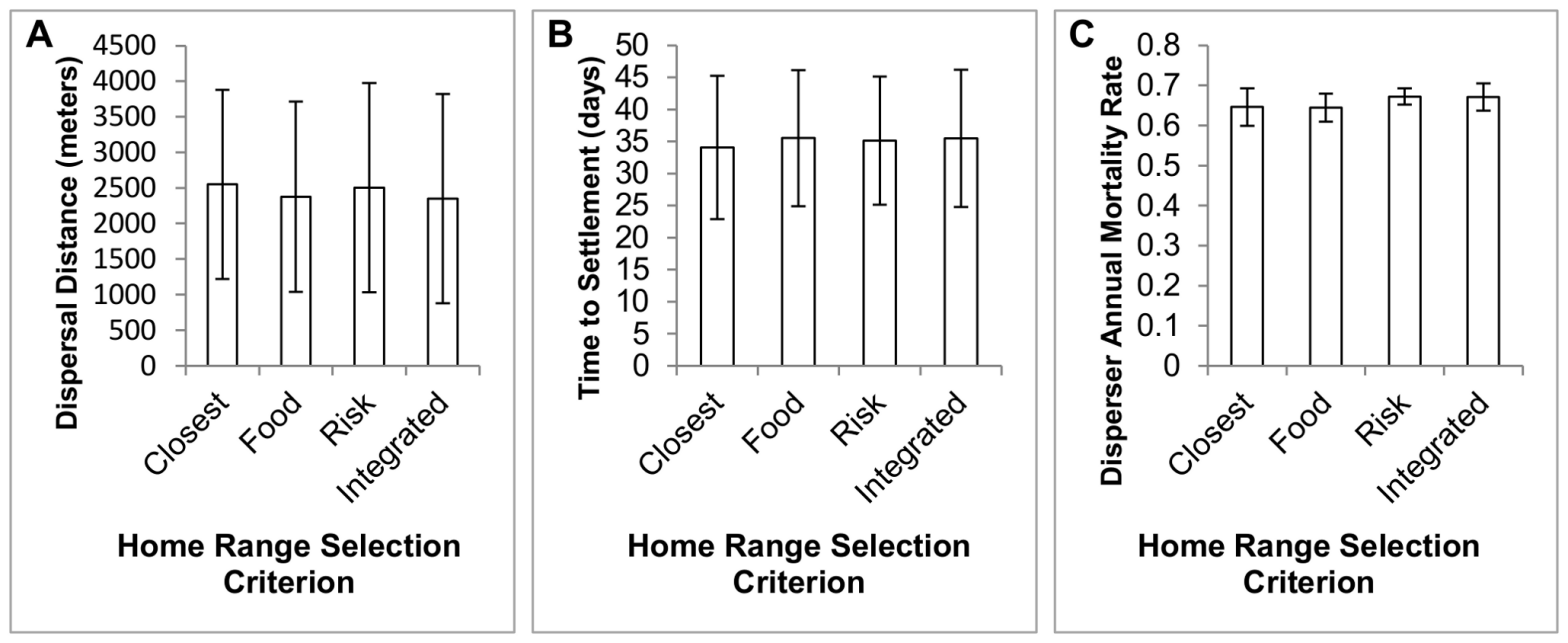
Effect of home range selection criterion on American martens. Mean(± 1 SD) A) dispersal distance B) settlement time and C) annual mortality for virtual marten home range selection scenarios. Simulations consisted of martens that selected home range locations based upon proximity (closest), proximity and food availability (food), proximity and predation risk (risk), or proximity, predation risk and food availability (integrated).

The criteria used for home-range center selection had little effect on any of the response variables of virtual animals. Mean dispersal distances were nearly identical for all four criteria (F_3, 8_ = 1.31, p = 0.338; Figure 4a). Similarly, settlement times were fairly constant across the home-range selection scenarios (F_3, 8_ = 1.69, p = 0.2450; Figure 4b). Finally, the mortality rates for simulations with the four criteria were nearly identical (F_3, 8_ = 0.56, p = 0.654; Figure 4c).

Overall, virtual martens in SEARCH simulations exhibited the same dispersal characteristics (distance, time and mortality rate) in response to a variety of home range selection rules. At first, these negative results appear inconsequential and such non-significant results are often overlooked [117]. This case study, however, highlights one of the major advantages of simulation models that have the capability of flexible levels of complexity. Models with features that can be turned on or off allow researchers to experimentally test the level of model complexity needed to adequately simulate the species in question [1]. In our case we found that more complex home-range selection criteria had no effect on the dispersal characteristics of virtual martens in Wisconsin. Therefore, future research on this study system could use simplified home-range selection rules (primarily the ‘closest’ home-range criterion) allowing for a simulation structure that only includes necessary complexity [19]. Of course this particular form of model simplification would not pertain to all cases, but the principle of refinement of model application through experimentation is a valuable asset that could be used in many implementations of SEARCH.

## Summary

We present SEARCH, a newly developed, spatially explicit, individual based model. SEARCH incorporates a high degree of behavioral complexity and allows for temporally dynamic landscapes. SEARCH is parameter-intensive which allows researchers to utilize all available data in parameterizing the model. However, SEARCH has the flexibility to allow users to “turn off” functions in the model when data for parameterization are unavailable (as would be the case for some component of the model in nearly every case). This functionality enables users to investigate when added behavioral complexity results in quantitatively different model outcomes. Thus, users can investigate the model's sensitivity to added complexity and evaluate the benefits and costs of incorporating behavioral complexity. Users are therefore able to optimize model functionality for the research question and population under study.

SEARCH is applicable to a number of species in a wide variety of systems though probably best suited for solitary mammals. It is a model that is ideal for simulating behaviorally complex populations with small abundances in a conservation setting. Furthermore, SEARCH allows researchers to simulate habitat and population manipulations that would be impractical in a field setting and offers that ability to project population dynamics into the future. There are a number of limitations to SEARCH, however. For example, the interaction between individuals in SEARCH is fairly rudimentary and the dynamic aspects of maps in SEARCH must be determined *a priori* (rather than as a response to model behavior). Furthermore, the breeding algorithms in SEARCH are not spatially explicit nor are they responsive to the state of the individual animal. Despite such shortcomings, SEARCH offers researchers a tool for investigating animal dispersal (and the subsequent population dynamics) that is not species specific but is capable of incorporating behavioral complexity not found in most comparable models. Thus the use of this tool has the potential to offer valuable insight into the role of the interplay between complex behavior and landscape configuration to animal population dynamics and management.

## Supporting Information

Figure S1 **SEARCH process schematic.** Process flow of SEARCH simulation (left) with detailed schematic of animal processes during dispersal (right).
(PDF)

Table S1 **Spatial parameters of raccoon simulations with values for each habitat type corresponding to the movement map, the risk map and the food map.**
(PDF)

Table S2 **Spatial parameters of chipmunk simulations with values for each habitat type corresponding to the movement map, the social map and the risk map.**
(PDF)

Table S3 **Spatial parameters of movement map for American marten simulations.**
(PDF)

Table S4 **Spatial parameters of food map for American marten simulations.**
(PDF)

Table S5 **Spatial parameters of risk map for American marten simulations.**
(PDF)

Table S6 **Modifier values for behavioral states of American martens with various levels of behavioral switching.**
(PDF)

References S1 **Citations for materials referenced in supporting information documents.**
(PDF)

Text S1 **Submodel descriptions.**
(PDF)

Text S2 **Technical documentation.**
(PDF)
