# Supplementary figures and images for: SEARCH: Spatially Explicit Animal Response to Composition of Habitat
Source: PLoS One. 2013 May 22;8(5):e64656. doi: 10.1371/journal.pone.0064656 (PMC3661500; doi:10.1371/journal.pone.0064656)

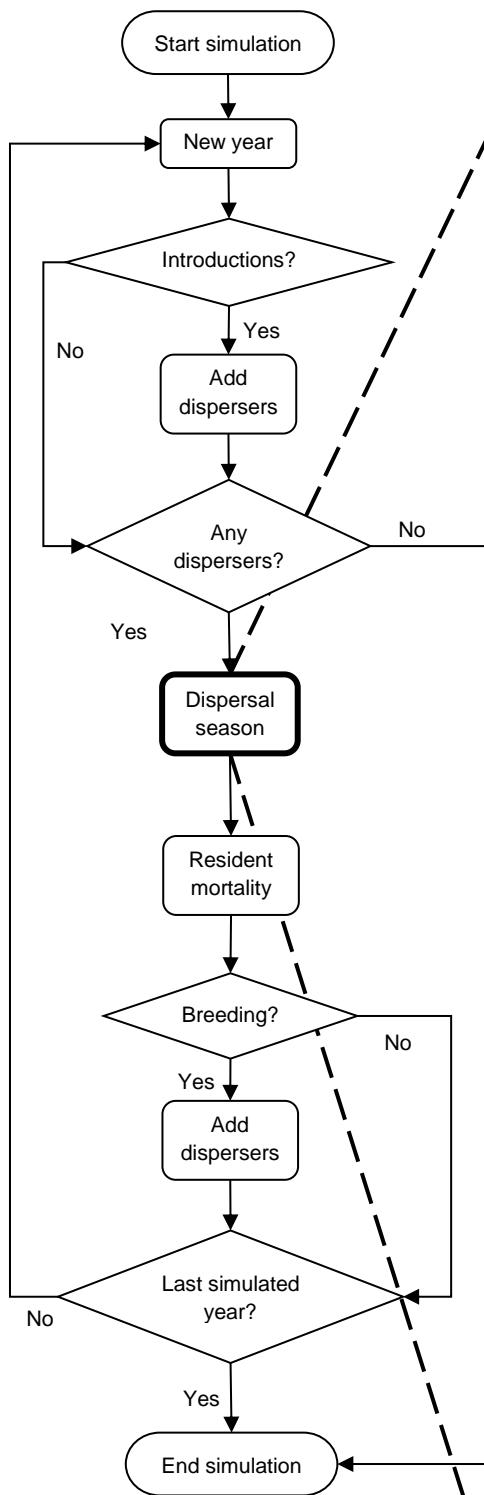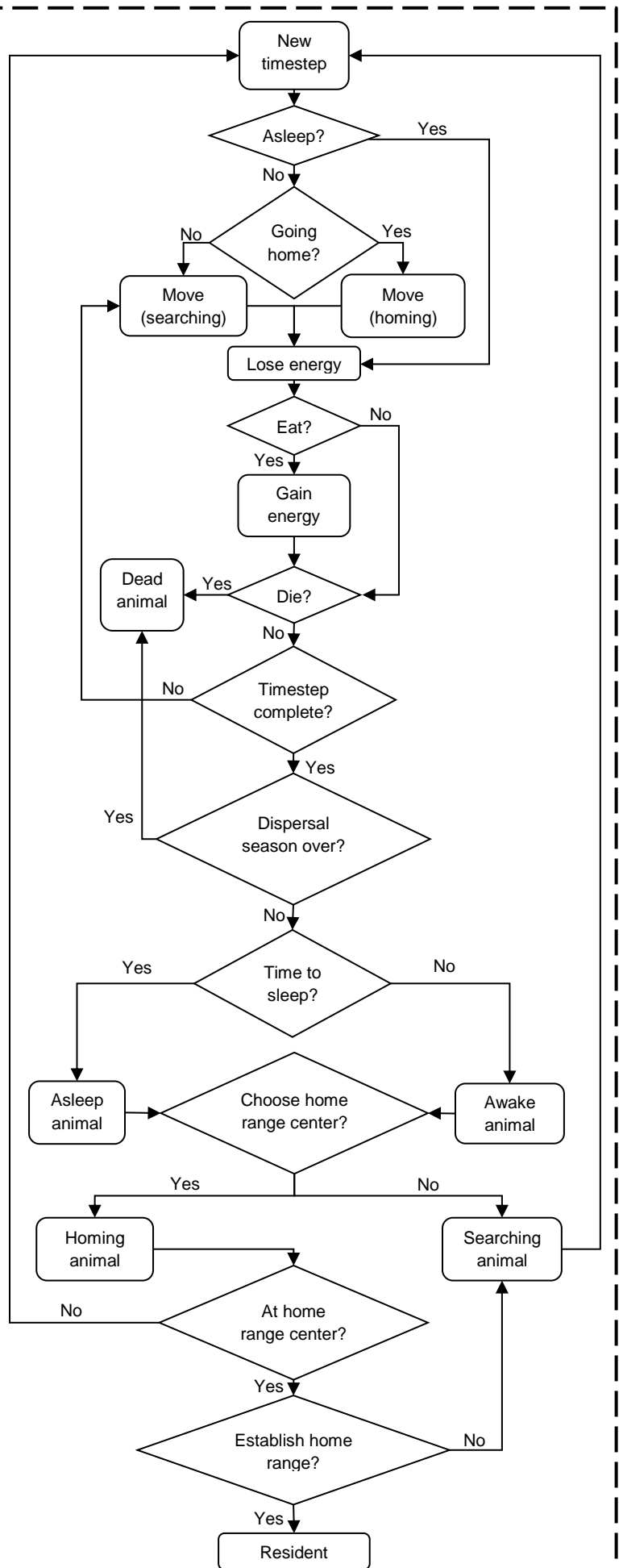

Supplement: Figure S1 — SEARCH process schematic. Process flow of SEARCH simulation (left) with detailed schematic of animal processes during dispersal (right). (PDF) [file pone.0064656.s001.pdf]
