# Supplementary material for: SEARCH: Spatially Explicit Animal Response to Composition of Habitat
Source: PLoS One. 2013 May 22;8(5):e64656. doi: 10.1371/journal.pone.0064656 (PMC3661500; doi:10.1371/journal.pone.0064656)
Supplement: Table S1 — Spatial parameters of raccoon simulations with values for each habitat type corresponding to the movement map, the risk map and the food map. (PDF) [file pone.0064656.s002.pdf]

Table S1 - Spatial parameters of raccoon simulations with values for each habitat type corresponding to the movement map, the risk map and the food map.

	**Movement**					**Risk**	**Food**		
**Habitat**	**MVL[a]**	**MSL[b]**	**Energy use**	**Crossing value**	**Percep. mod.[c]**	**Risk**	**Prob. capture**	**Size**	**SD**
Agriculture	0.2	238	3.33	6	1	0.00005	-	-	-
Base[d]	-	-	-	-	-	-	0.6	8.0625	0
High[d]	-	-	-	-	-	-	0.9	8.75	0
Low[d]	-	-	-	-	-	-	0.3	6	0
Corridor	0.56	70	3.33	2.8	1	0.000064	0.6	5	0
Forest	0.26	152	3.33	20	0.8	0.000038	0.65	5.41667	0
Grassland	0.56	155	3.33	2.8	1	0.000064	0.6	5	0
Road	0.92	163	3	1	1.3	0.01	0	0	0
Shrubland	0.56	45	3.33	2.8	1	0.000064	0.6	5	0
Urban	0.92	115	3.33	1	0.85	0.00008	0.2	6.04167	0
Water	0.92	15	5	0.1	0.5	0.05	0	0	0
Wetland	0.26	82	3.33	15	1.2	0.000038	0.7	5.20833	0
Source(s)	Beasley unpublished data		[121] - [130]	[53]	[62] - [66]	[56], [122], [131] - [137]			

[a] Mean vector length for correlated random walk
[b] Mean step length
[c] Perceptual window modifier value
[d] Simulations either used a static value for foraging probability in agricultural areas (base) or values corresponding to scarcity, superabundance and scarcity in those areas (high, low and high, respectively)
