# Supplementary material for: SEARCH: Spatially Explicit Animal Response to Composition of Habitat
Source: PLoS One. 2013 May 22;8(5):e64656. doi: 10.1371/journal.pone.0064656 (PMC3661500; doi:10.1371/journal.pone.0064656)
Supplement: Table S2 — Spatial parameters of chipmunk simulations with values for each habitat type corresponding to the movement map, the social map and the risk map. (PDF) [file pone.0064656.s003.pdf]

Table S2 - Spatial parameters of chipmunk simulations with values for each habitat type corresponding to the movement map, the social map and the risk map.

	Movement								Social	Risk		
**Habitat**	**MVL**[a]	**MSL**[b]	**Energy use**	**Crossing Value**				**Percep. mod.**[c]	**Suitability**	**Mortality risk**[d]		
				**None**[e]	**Base**[e]	**Day**[e]	**Night**[e]			**Base**[f]	**Day**[f]	**Night**[f]
Aspen	0.625	1	0	1	197.6	197.6	197.6	1	Suitable	7.16	7.16	7.16
Bog	0.6	1	0	1	199.5	99.8	299.3	1	Not suitable	7.10	14.2	0
Buildings	NA	NA	0	0	0	0	0	1	Not suitable	NA	NA	NA
Conifers	0.65	1	0	1	200.9	100.4	301.3	1	Not suitable	7.05	14.1	0
Grass	0.9	1	0	1	100	50	150	1	Not suitable	14.2	28.3	0
Hardwood	0.638	1	0	1	204.5	306.7	102.2	1	Suitable	6.92	0	13.9
Shrubs	0.613	1	0	1	134.8	67.4	202.3	1	Suitable	10.5	21.0	0
Water	NA	NA	0	0	0	0	0	1	Not suitable	NA	NA	NA
Source(s)	Zollner unpublished data								Zollner unpublished data	[88], Zollner unpublished data		

[a] Mean vector length for correlated random walk

[b] Mean step length

[c] Perceptual window modifier value

[d] Risk values multiplied by 10^6

[e] The response of chipmunks in each scenario to habitat boundaries was either null (‘None’ crossing value), spatial (‘Base’ crossing value), spatial and temporal (alternating ‘Day’ and ‘Night’ crossing values) or predictive (alternating ‘Day’ and ‘Night’ crossing values 1 hour before risk map swap)

[f] Simulations either used a static value for predation risk (base) or alternating diurnal and nocturnal values (day and night, respectively)
