# Supplementary material for: SEARCH: Spatially Explicit Animal Response to Composition of Habitat
Source: PLoS One. 2013 May 22;8(5):e64656. doi: 10.1371/journal.pone.0064656 (PMC3661500; doi:10.1371/journal.pone.0064656)
Supplement: Table S3 — Spatial parameters of movement map for American marten simulations. (PDF) [file pone.0064656.s004.pdf]

Table S3 – Spatial parameters of movement map for American marten simulations.

Habitat	MVL[a]	MSL[b]	Energy use	Crossing	Percep. mod.[c]
Move habitat A	0.9999	100	5	1.5	0.8
Move habitat B	1	20	1	10	0.4
Move habitat C	0.825	30	1.5	8	0.4
Move habitat D	0.85	40	2	7	0.4
Move habitat E	0.925	75	3.75	2	0.65
Move habitat F	0.95	55	2.75	5	0.55
Move habitat G	0.85	45	2.25	4	0.4
Move habitat H	0.975	88	4.4	1	0.65

[a] Mean vector length for correlated random walk
[b] Mean step length
[c] Perceptual window modifier value
