# Supplementary material for: SEARCH: Spatially Explicit Animal Response to Composition of Habitat
Source: PLoS One. 2013 May 22;8(5):e64656. doi: 10.1371/journal.pone.0064656 (PMC3661500; doi:10.1371/journal.pone.0064656)
Supplement: Table S4 — Spatial parameters of food map for American marten simulations. (PDF) [file pone.0064656.s005.pdf]

Table S4 - Spatial parameters of food map for American marten simulations.

Habitat	Prob. capture	Size	SD
Food habitat 1	0.0001	30	20
Food habitat 2	0.02	40	70
Food habitat 3	0.03	60	70
Food habitat 4	0.019	60	50
Food habitat 5	0.008	180	360
Food habitat 6	0.012	30	20
Food habitat 7	0.004	160	370
Food habitat 8	0.002	70	80
