# Supplementary material for: SEARCH: Spatially Explicit Animal Response to Composition of Habitat
Source: PLoS One. 2013 May 22;8(5):e64656. doi: 10.1371/journal.pone.0064656 (PMC3661500; doi:10.1371/journal.pone.0064656)
Supplement: Table S5 — Spatial parameters of risk map for American marten simulations. (PDF) [file pone.0064656.s006.pdf]

Table S5 - Spatial parameters of risk map for American marten simulations.

Habitat	Risk
Risk habitat R	0.000002
Risk habitat S	0.000006
Risk habitat T	0.000009
Risk habitat U	0.000038
Risk habitat V	0.00005
Risk habitat W	0.00015
Risk habitat X	0.0003
Risk habitat Y	0.03
Risk habitat Z	0.000001
