# Supplementary material for: SEARCH: Spatially Explicit Animal Response to Composition of Habitat
Source: PLoS One. 2013 May 22;8(5):e64656. doi: 10.1371/journal.pone.0064656 (PMC3661500; doi:10.1371/journal.pone.0064656)
Supplement: Table S6 — Modifier values for behavioral states of American martens with various levels of behavioral switching. (PDF) [file pone.0064656.s007.pdf]

Table S6 - Modifier values for behavioral states of American martens with various levels of behavioral switching.

	Modifier type[a]								
**Parameter**	**Base**	**Risky**	**Safe**	**Forage**	**Search**	**Risky-forage**	**Risky-search**	**Safe-forage**	**Safe-search**
Probability of food capture	1.00	1.00	1.00	2.00	0.50	2.00	0.50	2.00	0.50
Energy use	1.00	1.00	1.00	0.50	2.00	0.50	2.00	0.50	2.00
Mean step length	1.00	2.00	0.50	0.50	2.00	1.00	4.00	0.25	1.00
Mean vector length	1.00	1.00	1.00	0.50	2.00	0.50	2.00	0.50	2.00
Perceptual window	1.00	0.50	2.00	0.50	2.00	0.25	1.00	1.00	4.00
Probability of mortality	1.00	2.00	0.50	1.00	1.00	2.00	2.00	0.50	0.50

[a] Virtual animals were in either a safe or risky state and were either in searching or foraging mode.
