# Supplementary material for: SEARCH: Spatially Explicit Animal Response to Composition of Habitat
Source: PLoS One. 2013 May 22;8(5):e64656. doi: 10.1371/journal.pone.0064656 (PMC3661500; doi:10.1371/journal.pone.0064656)
Supplement: References S1 — Citations for materials referenced in supporting information documents. (PDF) [file pone.0064656.s008.pdf]

References S1. Citations for materials referenced in supporting information documents.

Citation numbers are a continuation of those from the main manuscript.

118 Kareiva PM, Shigesada N (1983) Analyzing insect movement as a correlated random walk. Oecologia 56: 234-238.

119 Berec L (2002) Techniques of spatially explicit individual-based models: construction, simulation, and mean-field analysis. Ecol Model 150: 55-81.

120 Bunimovich LA (1979) On the ergodic properties of nowhere dispersing billiards. Comm Math Phys 65: 295-312.

121 Mugaas JN, Broudy E, Seidensticker J (1983) Metabolism of male raccoons, *Procyon lotor*, in summer. Am Zool 23: 937A.

122 Teubner VA, Barrett GW (1983) Bioenergetics of captive raccoons. J Wildl Manage 47: 272-274.

123 Mugaas JN, Mahlke KP, Broudy E, Meyer A, Seidensticker J (1984) Metabolism of raccoons, *Procyon lotor*, in winter and summer. Am Zool 24: 89A.

124 Chevalier CD (1987) Comparative thermoregulation in tropical procyonids. Am Zool 27: 146A.

125 Mugaas JN, Seidensticker J, Mahlke-Johnson KP (1993) Metabolic adaptation to climate and distribution of the raccoon *Procyon lotor* and other procyonidae. Smithson Contrib Zool 542: 1-34.

126 McNab BK (1995) Energy expenditure and conservation in frugivorous and mixed-diet carnivorans. J Mamm 76: 206-222.

127 Willmer P, Stone G, Johnston I (2000) Environmental physiology of animals. Oxford: Blackwell Publishing. 644 p.

128 Nagy KA (1987) Field metabolic rate and food requirement scaling in mammals and birds. Ecol Monogr 57: 111-128.

129 Koteja P (1991) On the relation between basal and field metabolic rates in birds and mammals. Funct Ecol 5: 56-64.

130 Ricklefs RE, Konarzewski M, Daan S (1996) The relationship between basal metabolic rate and daily energy expenditure in birds and mammals. Am Nat 147: 1047-1071.

131 Hamilton WJ Jr (1936) The food and breeding habits of the raccoon. Ohio J Sci 36: 131-140.

132 Giles LW (1939) Fall food habits of the raccoon in central Iowa. J Mamm 20: 68-70.

133 Baker RH, Newman CC, Wilke F (1945) Food habits of the raccoon in eastern Texas. J Wildl Manage 9: 45-48.

134 Yeager LE, Elder WH (1945) Pre- and post-hunting season foods of raccoons on an Illinois goose refuge. J Wildl Manage 9: 48-56.

135 Schoonover LJ, Marshall WH (1951) Food habits of the raccoon (*Procyon lotor hirtus*) in north-central Minnesota. J Mamm 32: 422-428.

136 Johnson AS (1970) Biology of the raccoon (*Procyon lotor varius* Nelson and Goldman) in Alabama. Auburn: Auburn University Agricultural Experiment Station Bulletin 148 p.

137 Hoffman CO (1979) Weights of suburban raccoons in southwestern Ohio. Ohio J Sci 79: 139-142.

138 Topping CJ, Høye TT, Olesen CR (2010) Opening the black box – development, testing and documentation of a mechanistically rich agent-based model. Ecol Model 221: 245-255.
