# Supplementary material for: SEARCH: Spatially Explicit Animal Response to Composition of Habitat
Source: PLoS One. 2013 May 22;8(5):e64656. doi: 10.1371/journal.pone.0064656 (PMC3661500; doi:10.1371/journal.pone.0064656)
Supplement: Text S1 — Submodel descriptions. (PDF) [file pone.0064656.s009.pdf]

Text S1. Supplementary submodel descriptions.

S.1. Movement

Movement in SEARCH is characterized by the direction of movement, speed of movement and the response to boundaries. Additionally, an animal’s perception of areas it has traversed is controlled by the movement maps.

S.1.1. Direction

Animals in SEARCH have two modes of movement – non-directed and directed movement. In non-directed movement (used before an animal selects a potential home-range center) animals follow a correlated random walk [118] where direction of movement is correlated with the previous movement direction (base direction is the bearing of the previous step). Deviations in bearing from the base direction are drawn from a wrapped Cauchy distribution [43], [44] based on the mean vector length value of the animal’s location (along with applicable modifiers – see section S.6 for more details). Directed movement is the same as non-directed movement except that the base orientation of movement is redirected towards the chosen home-range center at the beginning of each time-step and is independent of previous steps.

S.1.2. Crossing

When animals encounter a boundary on the move map they either cross that boundary into a new polygon or reflect off the boundary and remain in their current polygon. This decision is made by comparing the crossing values of each polygon and

calculating a probability of crossing $p = n/c$, where p equals the probability of crossing the boundary, n equals the rank of the new location, and c equals the rank of the current location. A random number (r) is generated from a uniform distribution between 0 and 1. The animal will move into the new habitat if $p > r$, otherwise the animal will reflect 180º at the boundary and continue in the current habitat type for the remainder of the time-step (or until another boundary is encountered). Since the value of r is 0-1, if the new polygon has a higher value than the current polygon the animal will always cross. However, if the current polygon has a higher value the animal will still have some probability (<1) of crossing.

Animals that encounter the edge of the map do not respond to that boundary and move outside of the map. SEARCH employs an absorptive boundary [119] so that animals that move outside the extent of the map are removed from the simulation and are treated the same as dead dispersers. Alternatively, a reflective boundary [119] can be implemented in SEARCH by enclosing the outside edge of the move map with a polygon with crossing value 0.

S.1.3. Speed

Animal speed is based upon the mean step length of a particular habitat (along with applicable modifiers – see section S.6 for more details). If no boundary is encountered on the movement map, an animal will move the designated step length in the direction of orientation.

If a boundary is encountered during a step an animal will move to that boundary and decide whether or not to cross (see section S.1.2 for more details). If the boundary

is crossed the animal will move the remaining proportion of the step in the new polygon based on the step length of that polygon (i.e. (1 – first proportion)*step length of new polygon). If another boundary is encountered during this portion of the step, the process is repeated.

If a boundary is encountered and the animal does not cross, it will reflect off the boundary (i.e. turn 180°) and move the remaining proportion of the step in that direction. If another boundary is encountered during this portion of the step, the process is repeated.

S.1.4. Perceptual Window

The area an animal observes and remembers (stored in individual memory maps) during dispersal is a function of its perceptual window during movement. The baseline perceptual value for all dispersers is specified by the user. This value is modified based on the perception values of the movement map and by all applicable modifiers (see section S.6 for more details). A stadium (rectangle with height 2*r*, length *n* and semicircles on each end with radius *r*) [120] with a radius of this distance is produced along the line-segments connecting the animal's travel path. The animal's memory is the total area within these stadia for all time-steps.

S.2. Foraging

In SEARCH dispersers gain and lose energy based on their ability to forage successfully, the amount of energy they gain during a successful foraging bout, the amount of energy they use during movement and their energy limits.

S.2.1. Energy Gain

Virtual animals in SEARCH attempt to forage during each full or partial time-step. Animals gain energy when they successfully capture prey. The probability of successfully capturing prey is controlled by the location of an animal relative to the food map (multiplied by the proportion of the step completed within a polygon along with applicable modifiers – see sections S.1 and S.6 for more details). A random number is drawn and if it exceeds the animal's probability of successfully foraging the animal does not gain any energy. If the random value is less than or equal to the foraging probability the animal will gain energy. The animal's energy reserves will increase based on a random number drawn from a normal distribution with mean (± SD) derived from the values of the food map at the animal's location (unless the energy maximum is reached – see section S.2.3 for details). If the value drawn is a negative value, energy gained will equal 0 and the animal will not gain energy.

S.2.2. Energy Loss

During each partial and full time-step animals lose energy due to metabolic loss. The base value of energy loss is determined by the animal's location on the move map (and differs in different areas due to energetic costs associated with different habitat types). The animal's energy reserves decrease this amount (multiplied by the proportion of the step completed along with applicable modifiers – see sections S.1 and S.6 for more details).

S.2.3. Energy Limits

Animals are assigned energy maximums, energy minimums and a search/forage trigger. Energy levels are not allowed to exceed the energy maximum. If an energy gain were to cause the energy level to exceed maximum it is set to the maximum level. Similarly, energy levels are not allowed to drop below the minimum for dispersers. When energy losses cause the level to fall below the minimum the animal dies of starvation and is recorded as such in its text file. The search/forage trigger is a set energy level between the minimum and maximum that controls animal behavior. When the energy level is below the trigger, the animal is in foraging mode (controlled by the safe-foraging and risky-foraging modifiers – see section S.6 for more details). When the energy level exceeds the trigger, the animal is in searching mode (controlled by the safe-searching and risky-searching modifiers – see section S.6 for more details).

S.3. Mortality

All individual animals in SEARCH are subject to mortality probabilities. Dispersers that die stop moving and cannot establish home ranges. Residents that die have their home range removed from the social map and are not able to breed or occupy suitable habitat during subsequent years.

S.3.1. Disperser Mortality

Juvenile dispersers are subject to a number of mortality fates. They can die of starvation (see section S.2 for details), they can die of winter kill if they fail to establish a

home range during the dispersal season, or they can die of predation. Additionally, animals that move outside the spatial extent of the map are removed and treated as dead animals (see section S.3 for details) but a reflective outer boundary can be parameterized (see section S.1.2 for details). In SEARCH, predation mortality serves to encompass all mortality causes other than those specified (i.e. it includes risk of death from disease, harvest, predation, etc.). Predation risk for a time-step is based on the mortality risk for a specific location, the proportion of a time-step taken and all applicable modifiers (see sections S.1 and S.6 for more details). The morality risk (R) for an animal is $1 – [(1 – JM)^P]$ where J is the product of all applicable modifiers, M is the probability of mortality based on location and P is the proportion of a time-step completed during the movement segment (if no boundaries are encountered P=1). If a drawn random number is below R, the animal dies.

S.3.2. Resident Mortality

Unlike dispersers, there are two types of mortality probabilities for residents and both are aspatial. Residents are subject to user-defined per time-step mortality during the dispersal season and an inter-dispersal mortality probability. For each time-step during the dispersal season and once during the inter-dispersal season a random number is drawn for each resident. If that value is below the per time-step resident mortality probability or the inter-dispersal mortality, the animal dies.

Residents may also die due to a social map swap. When a social map is replaced during a simulation residents attempt to reestablish a home range in their current location (see section S.4.3 for details). If they are unable to do this (due to lack

of suitable and unoccupied area) they die. Residents are unable to attempt home-range reestablishment anywhere but their previous home-range center.

S.4. Home-Range Establishment

Dispersers in SEARCH begin at the center of their natal home range and attempt to move throughout the landscape and establish their own home range. During the searching process they exceed some user-defined threshold and choose a potential home-range location from their memory map. Then they return to the selected potential home-range center and attempt to establish a home range. Dispersers that do not establish a home range by the end of the dispersal season die of winter kill.

S.4.1. Trigger

Dispersers in SEARCH can be triggered to begin the home-range establishment process in one of two ways (depending on user specification). As the animal moves it keeps track of the end points of each active time-step. After a specified number of active steps have been taken (i.e. number of points exceeds threshold) or after a specified number of suitable and unoccupied (by the same-sex) sites have been visited (i.e. number of points in suitable and unoccupied areas exceeds threshold) an animal will choose a potential home-range center.

S.4.2. Home-Range Center Selection

Once animals have exceeded the home-range trigger criterion, they choose a point for a possible home-range center. To do this the animal constructs a list of all

suitable sites that were visited, unoccupied by a same sex resident and within a continuous area perceived by the disperser that was greater than 1/10 the minimum home-range area for that animal (if no points fit these criteria the animal continues dispersing until one or more do). The distance between every suitable point and the animal's current location is calculated. Each site is assigned a raw rank value based on the decision criterion in use. For the "closest" criterion, only the distance to a site is considered such that each site is assigned a value of $\sqrt[n]{d}$ where *d* is the distance to the site and *n* is the user-specified distance weight factor for that sex. For all criteria, site proximity can be negated by making *n* large. For the "least risk" criterion, both distance and perceived risk are taken into account such that each site is assigned a value of $(1-r)/\sqrt[n]{d}$ where *d* is the distance to the site, *n* is the distance weight factor for that animal and *r* is the perceived risk of mortality at that site at the time the animal visited it. With the "best food" criterion, both distance and probability of food capture are considered such that sites are valued as $f/\sqrt[n]{d}$ where *d* is the distance to the site, *n* is the distance weight factor and *f* is the probability of food capture at that site at the time the animal visited it. Finally, the "integrated" criterion considers distance, risk and food capture. To normalize the scale for food and risk values, each site uses a standardized value of food and risk for ranking. For food capture $f_{std} = f / f_{max}$ where f_{std} is the standardized food value, *f* is the probability of food capture at a site and f_{max} is the maximum probability of food capture experienced by that animal during dispersal. Similarly, risk is standardized by $r_{std} = r / r_{max}$ where r_{std} is the standardized risk value, *r* is the probability of mortality at a site and r_{max} is the maximum mortality probability experienced by that animal during dispersal. To rank locations under the integrated

criterion, each site gets a value of $[f_{std} + (1 - r_{std})]/\sqrt[n]{d}$ where d is the distance to the site, n is the distance weight factor, and f_{std} and r_{std} are the standardized values for food capture and risk, respectively. Regardless of the home-range selection criterion used, all raw ranks are divided by their sum and sequentially added so that each site gets a unique interval from 0 to 1, the range of which describes its probability of being chosen. A random number is selected and the interval into which that number falls determines which site is chosen as the home-range center. The animal then attempts to return to that site using the directed mover (see section S.1.1 for more detail). Once an animal has chosen a home-range center, it attempts to move back to that location. When the distance between the animal and that site is less than the animal's perceptual window at that location, the animal is assumed to have arrived at the desired location and attempts to establish a home range at that site.

S.4.3. Home-Range Generation

In order to establish a home range, the animal must create a potential polygon after generating 30 points. To do this the animal selects a random bearing from the home-range center. A point is created on this bearing at a distance of h meters from the home-range center where $h = j * r * s$ where j is a random number drawn from a normal distribution of mean 1.2 and standard deviation of 0.1, r is the radius of a circle with the size of the animal's minimum home range ($r = \sqrt{(HR_{min}/\pi)}$) and s is the stretch factor of 1.0. This is replicated 30 times with 30 random bearings and these points are connected in counterclockwise order to form a polygon. All areas that are unsuitable or occupied by the same sex are removed from this polygon. If the remaining area is greater than

the minimum home-range area for the animal, this area becomes that animal's home range. If the area is less than the minimum home-range area, the points are discarded and 30 new points are created using the same algorithm with a 0.1 increase in the stretch factor. These steps are repeated up to 10 times. If after 10 attempts the animal fails to successfully establish a home range it selects a new home-range center using the same process as above and repeats the process. If a home range is established, the social map changes to reflect the change in occupancy and the disperser becomes a resident subject only to resident functions.

S.5. Creating Animals

New animals can be created in SEARCH in three ways. First, residents can be generated by using a social map in the first simulation year that has resident home ranges delineated. Second, dispersers can be created by implementing release maps at any time in the simulation. These point maps specify the location, sex, and number of released animals. Finally, animals can be created via reproduction of resident females. All dispersers have the same initial values for energy level, all begin in the safe-searching mode and all have the same relative activity periods.

S.5.1. Initial Residents

In the first year of the simulation it is possible to create initial residents to populate the social map. Residents can be created in this way by having polygons that have unique alphanumeric labels for male or female occupancy. These resident home ranges are given a new unique number within SEARCH (to avoid confusion) and are

treated as all other residents in the simulation. Initial residents are subject to stochastic breeding beginning in the first inter-dispersal period.

S.5.2. Releases

Dispersers can be created through the use of the release map. At the beginning of the simulation and again at any specified time during the simulation a release map can be inserted which dictates the location and sex of released animals. These animals begin dispersing immediately following release and behave just as other dispersers.

S.5.3. Reproduction

SEARCH also creates new dispersers through resident breeding. During the inter-dispersal period each surviving female resident has the chance to breed. For each female, if the random number drawn is below the value for the probability of pregnancy, the animal becomes pregnant; otherwise the animal does not breed that year but is eligible to breed again in subsequent years assuming it survives. For pregnant females, the number of offspring is an integer drawn from a distribution based on the mean and standard deviation of litter size input by the user (if the number drawn is negative, no animals are born). The sex for each offspring is decided based on the probability of young being female (as specified by the user). If the random number drawn is below this value, the offspring will be female; otherwise it will be male. Offspring begin dispersal at the start of the next dispersal season and begin at the center of their mother’s home range.

S.6. Modifiers

Modifiers are real numbers with a multiplicative effect on various aspects of dispersal activity. These effects are incorporated to reflect differences in behavior of certain individuals (i.e. males or females) or during certain circumstances (i.e. during particular times of day or while animals are in certain behavioral states). All modifiers are capable of affecting the probability of capturing food, energy use per time-step, mean step length, mean vector length, perceptual window and mortality probability. Modifiers can be any non-negative real number or be set to 1 for no effective impact. Modifiers can be set based on sex (male or female), behavior (any of the 4 behavioral states – risky-search, risky-forage, safe-search, safe-forage), and time (at both a cyclic daily scale and a seasonal scale) but only alter values for appropriate animals and situations. For instance, male modifier values would only influence values for male animals, risky-foraging modifiers would only be used when an animal was in the risky-foraging behavior, and a temporal modifier would only take effect during the time in which it was specified. All modifiers work in conjunction with one another in a multiplicative fashion such that many modifiers could be in effect at the same time for an animal and it is possible to have multiple versions of each temporal modifier (e.g. an hourly modifier for 6am-8am, another for 8am-2pm, and another for 2pm-6am all in the same run).

S.7. Activity and Behavioral States

In addition to behavior based on energy reserves (i.e. searching vs. foraging – see section S.2.3 for details), animals in SEARCH also change their behavior in response to daily activity periods and perceived risk of mortality.

S.7.1. Activity Periods

At any time during the dispersal season, each disperser in SEARCH is either active or resting. Resting animals do not move or set up home ranges but are subject to energy losses, energy gains, and mortality. Animal activity periods are set by the user by defining the mean and standard deviation for the number of hours spent in either state. All mean activity periods in a single daily cycle must sum to 24 but can include many active and resting periods (e.g. active 4 h, resting 6.5 h, active 8.5 h, and resting 5 h). The first activity state is always active. Animals begin their cycle of activity from the first time-step after they are created (first time-step of first day for offspring, first time-step after release for released animals). After that the length of time in a particular activity state is based on the random number drawn from the distribution (mean ± SD) specified by the user. This means that the total of all active and resting periods could exceed or fall below 24 hours within a daily cycle. Activity periods (mean and SD) are applied to all dispersers and do not change during the simulation.

S.7.2. Behavioral States

Dispersers can change their behavior in response to perceived risk of mortality to either a safe or risky behavior (that in conjunction with the searching or foraging behavior determines which behavioral modifier takes effect). Default animal activity is risky but may change to safe if the animal perceives a heightened risk of mortality and may change back again after the animal returns to a feeling of relative safety. This is simulated when a number drawn from a uniform distribution (r) falls between PD and $[PD + M_s + M_r]$, where PD is the probability of depredation for the current location, M_s is input by the user to identify the probability that an animal will change to safe behavior,

and M_r is input by the user to identify the probability that an animal will return to risky behavior (both M_s and M_r are aspatial). The animal will enter a safe behavior if r is between PD and [$PD + M_s$] and will act in a risky behavior at subsequent steps if r is between [$PD + M_s$] and [$PD + M_s + M_r$]. If r is greater than [$PD + M_s + M_r$] the animal will remain in its previous activity mode.
