# Supplementary material for: SEARCH: Spatially Explicit Animal Response to Composition of Habitat
Source: PLoS One. 2013 May 22;8(5):e64656. doi: 10.1371/journal.pone.0064656 (PMC3661500; doi:10.1371/journal.pone.0064656)
Supplement: Text S2 — Technical documentation. (PDF) [file pone.0064656.s010.pdf]

Text S2. Supplementary technical documentation (ODdox).

Model Overview

The following documentation is intended to offer more technical description of SEARCH than is provided in the description manuscript or sub-model supplementary material. This document generally follows the ODdox framework [34, 138] as a means of detailing large agent-based models. Therefore, the following sections include concise descriptions of the ODD topics (covered in more detail in main manuscript) with reference to the appropriate classes in the code which are detailed in the Doxygen documentation (van Heesch 1997) available at:

http://paz-search.googlecode.com/svn/wiki/doxygen/index.html

SEARCH was not designed with the intent of such documentation; therefore some aspects of the technical documents may provide informal or incomplete descriptions.

Purpose

SEARCH simulates the dispersal and home-range establishment of animals as they respond to GIS layers dictating animal movement, foraging, risk of predation, and the social landscape. Output from SEARCH provides information on the characteristics of animal dispersal and the associated emergent population-level attributes. SEARCH can be used to simulate a variety of species and utilizes research from disparate fields such as animal movement, foraging ecology, and physiology to parameterize the model.

State Variables and Scales

Dispersers in SEARCH are characterized by a unique number, sex, weight, perception, activity mode, behavioral state, and location. State variables for residents include animal number, sex, and home-range location. See Animal class

Dispersers interact with the environment by responding to habitat characteristics represented in four vector GIS maps. Polygon values contained in these maps drive animal movement, foraging, mortality and home-range establishment by dictating correlated random walk movement parameters, the probability of acquiring prey and size of prey, the probability of being killed, and the suitability for home-range establishment and whether or not a location is currently occupied by a resident animal, respectively. See Map and MapManager classes.

Time periods in SEARCH are discrete and the spatial scales employed by SEARCH follow the resolution and extent of user-input GIS maps.

Process Overview and Scheduling

During SEARCH simulations, animals traverse a virtual landscape composed of GIS maps to model animal behavior. The initial landscape is populated by adult residents and/or released juveniles from the social and release maps, respectively. As animals move throughout the landscape, all four maps are queried during each time-step by each animal. See Mover class. After each animal movement segment, the location, energetic reserves, and behavioral states are updated for that individual. Animals move, forage, die and establish home ranges according to habitat and species parameters. During a time-step, each animal completes every action for that time-step in sequential order based on animal number assigned geographically at the beginning of each year. See Animal and AnimalManager classes.

Once a user-defined threshold for number of steps taken or number of sites visited is exceeded, animals select a site for a possible home-range center from areas searched during dispersal. Virtual animals then move to that site, and attempt to

establish a home range but may continue dispersing if they fail to locate a site with unoccupied suitable habitat. See HomeRangeFinder and HomeRangeBuilder classes. Animals become residents once a home range is established, but die if a home range is not established during the dispersal period. During the inter-dispersal period, residents are subject to random mortality and females stochastically give birth to young which disperse the following dispersal season. See Resident class.

Design Concepts

Objectives

In SEARCH each disperser's objective is to establish a home range before the end of the dispersal period. The effectiveness of dispersers in SEARCH is dependent upon user parameterization.

Adaptation

Simulated animals make decisions in response to the environment and change behavior based on their individual experiences. In SEARCH virtual animals respond to perceived danger or low energy reserves by changing their behavioral state to respond to conditions. See AnimalAttributes class.

Sensing

Animals in SEARCH are able to detect information in their environment and respond to that information accordingly. During movement, virtual animals sense the predation pressure, foraging resources, habitat quality and the suitability of habitat for home range establishment along with the presence of resident animals around them. Animals maintain a memory of the habitat as they perceived it during dispersal and update their memory when a site is revisited. See MemoryMap class.

Stochasticity

Nearly every action taken by animals in SEARCH is probabilistic including movement, foraging, mortality, activity patterns, home-range establishment and breeding.

Emergence

Population-level patterns emerge based upon the behavior and interactions of the individual animals in response to the spatio-temporal configuration of habitat and conspecific residents. These emergent properties develop from the interaction of behavioral parameters, individual variation, interaction between individuals and stochasticity.

Observation

During SEARCH simulations, data on the behavioral state and fate of each individual are produced during every time-step. Output includes GIS maps of animal memories and home ranges and text files for disperser attributes. See OutPutManage class.

Details

Initialization

Animals in SEARCH can be created via introductions, breeding or both. See SimulationManager and InitialAnimalAttributes classes. At the beginning of each simulation an empty memory map is created for each individual. When animals begin selecting potential home-range centers, this map is used to eliminate all points in areas perceived as unsuitable or occupied. For each animal a text file is also created that records the animal’s conditions for all state variables during each time-step.

Input

Maps

Virtual animals in SEARCH respond to the user-specified parameters of five GIS maps. These map types include 4 polygon maps (movement, food, risk and suitability) and 1 point map (assigning location and number of released animals). The landscape in SEARCH is dynamic in that any of the 5 GIS layers can be replaced at any time during the simulation with a map with different parameters and/or spatial configuration. See Map and MapManager classes.

Species Attributes

Dispersers in SEARCH are responsive to a number of user-defined parameters describing their energetics, activity patterns, behavioral states and activity modes. See Animal and AnimalManager classes.

Home-Range Attributes

Animals in SEARCH disperse until they are triggered to look for a home range. At that point they attempt to move back to a chosen site and establish a home-range polygon on the social map. See HomeRangeFinder and HomeRangeBuilder classes.

Modifiers

In SEARCH, the user can modify the baseline parameters based upon sex, behavioral state and any number of temporal modifiers. See Modifier class for detail.

Resident Attributes

Residents in SEARCH are subject to mortality (during both dispersal and interdispersal seasons) and females can produce young. See Resident class for more detail.
